# Long-Term Effects of Preterm Birth on Children’s Brain Structure: An Analysis of the Adolescent Brain Cognitive Development (ABCD) Study

**DOI:** 10.1523/ENEURO.0196-22.2023

**Published:** 2023-06-07

**Authors:** Niloy Nath, Winnica Beltrano, Logan Haynes, Deborah Dewey, Signe Bray

**Affiliations:** 1 Child and Adolescent Imaging Research (CAIR) Program, University of Calgary, Calgary, Alberta T2N 1N4, Canada; 2 Alberta Children’s Hospital Research Institute, University of Calgary, Calgary, Alberta T2N 1N4, Canada; 3 Hotchkiss Brain Institute, University of Calgary, Calgary, Alberta T2N 1N4, Canada; 4 Department of Pediatrics, Cumming School of Medicine, Calgary, Alberta T2N 4N1, Canada; 5 Community Health Sciences, Cumming School of Medicine, Calgary, Alberta T2N 4N1, Canada; 6 Department of Radiology, Cumming School of Medicine, Calgary, Alberta T2N 4N1, Canada

**Keywords:** birthweight, cortical structure, MRI, neurodevelopment, preterm birth, subcortical structure

## Abstract

Approximately 10% of births are preterm [PTB; <37 weeks gestational age (GA)], which confers risk for cognitive, behavioral, and mental health challenges. Using the large and relatively diverse (i.e., designed to reflect sociodemographic variation in the United States population) Adolescent Brain Cognitive Development Study (ABCD Study), we characterized the impact of PTB on brain structure in middle-late childhood (9–10 years). The ABCD sample covers the GA spectrum, and the large sample size (∼11,500) permits consideration of how associations between PTB and brain structure are impacted by GA, sex, birthweight, and analytic choices such as controlling for total brain size. We found a pattern of relative cortical thinning in temporoparietal and dorsal prefrontal regions and thickening of medial prefrontal and occipital regions in PTB compared with children born full term (≥37 weeks GA). This pattern was apparent when controlling for mean thickness and when considering moderate (>32 and <37 weeks GA) and very PTB (≤32 weeks GA) separately, relative to full term birth. Surface area (SA) and subcortical volumes showed reductions in PTB children that were largely attenuated when controlling for brain size. Effects on cortical thickness (CT) and surface area were partially mediated by birthweight. Although boys are at increased risk for adverse outcomes following PTB, there was limited evidence of sex differences of PTB effects. Finally, cortical thickness effects estimated in a “discovery” sample (*N* = 7528) predicted GA in a holdout “replication” sample (*N* = 2139). Our findings help to clarify the effects of PTB on brain structure into late childhood across the GA spectrum.

## Significance Statement

Preterm birth (PTB) affects ∼10% of children and increases the risk of neurodevelopmental and mental health challenges. Here, we examined long-term effects of PTB on brain structure in middle-late childhood in the large and relatively diverse Adolescent Brain Cognitive Development (ABCD) sample. We further assessed the influence of gestational age, sex, birthweight, controlling for brain size and data quality. Our findings replicate a pattern of occipitotemporal and dorsal prefrontal cortical thinning in PTB that was seen in both moderate preterm and very preterm relative to full-term birth. Effects were similar in males and females and partially mediated by birthweight. Our findings suggest that community-recruited children born preterm show a pattern of structural alterations on a continuum that relates to gestational age and birthweight.

## Introduction

An estimated 10% of infants are born preterm (<37 weeks gestational age; GA; Chawanpaiboon et al., 2019), with 1–5% very preterm (<32 weeks GA) or very low birth weight (≤1500 g), increasing risk for neurodevelopmental, cognitive, and mental health challenges (Hack et al., 2009; Johnson and Marlow, 2011; Vanes et al., 2022). Characterizing long-term effects of preterm birth (PTB) on brain structure could provide insights into the neural basis of the “transdiagnostic biological vulnerability to psychopathology” in PTB (Vanes et al., 2022).

Studies in children (Hasler et al., 2020; Mürner-Lavanchy et al., 2014; Sripada et al., 2018; Vandewouw et al., 2020), adolescents (Martinussen et al., 2005; Nagy et al., 2011), and adults (Bjuland et al., 2013; Pascoe et al., 2019; Rimol et al., 2019; Schmitz‐Koep et al., 2020) have reported regions of thinner cortex in individuals born preterm, with temporal and occipitoparietal regions frequently identified. Some studies have also reported thicker cortex in occipital (Kelly et al., 2014; Sripada et al., 2018) and prefrontal/orbitofrontal (Sølsnes et al., 2015; Sripada et al., 2018) regions. Relatively smaller surface area (SA) in temporo-parietal and dorsal visual regions have been associated with PTB (Skranes et al., 2013; Grunewaldt et al., 2014; Sølsnes et al., 2015; Zhang et al., 2015). Reduced SA has also been found in anterior temporal (Sølsnes et al., 2015), inferior frontal (Sølsnes et al., 2015), and ventral visual (Sripada et al., 2018; Hasler et al., 2020) regions. However, some studies have reported no group differences in SA (Mürner-Lavanchy et al., 2018; Young et al., 2020).

Beyond the cortex, enlarged ventricles have been reported in radiologic review (Hedderich et al., 2021) and volumetric studies (Cooke and Abernethy, 1999; Stewart et al., 1999; Nosarti et al., 2002; Kesler et al., 2004). Periventricular injury has been linked with neuronal loss in the thalamus and basal ganglia (Volpe, 2009), and MRI studies have shown volumetric reductions in thalamus (Nosarti et al., 2002; Lax et al., 2013; Brumbaugh et al., 2016; Sølsnes et al., 2016; Botellero et al., 2017; Sripada et al., 2018; Córcoles-Parada et al., 2019) and basal ganglia (Nagy et al., 2009; Botellero et al., 2017; Karolis et al., 2017; Schmitz-Koep et al., 2021). Cerebellar hemorrhage also occurs in a subset of infants born preterm (Garfinkle et al., 2020) and gliosis and cell loss in the dentate nucleus and cerebellar cortex of preterm infants has been reported (Pierson et al., 2007) believed to be reflected in cerebellar volume reduction (Allin et al., 2001; Parker et al., 2008).

Despite some similar findings, there are differences across studies in regions identified as altered in PTB. Methodological differences that may contribute include sample size, sensitivity related to 1.5T versus 3T MRI, population (defined based on gestational age or birthweight, presence of comorbidities), sociodemographic differences between preterm and control groups (Thompson et al., 2020), and software pipeline and analyses. While some studies have investigated relative size differences controlling for total brain, or intracranial, volume (Karolis et al., 2017; Vandewouw et al., 2020), many studies report absolute differences (where regional differences may reflect diffuse effects of overall smaller brain size), increasing the difficulty of synthesizing results. Further, birthweight has been shown to have long-term associations with brain structure in full-term born (FTB) samples (Walhovd et al., 2012) and therefore could mediate associations between preterm birth and brain structure.

The Adolescent Brain Cognitive Development Study (ABCD Study) presents a unique opportunity to characterize long-term impacts of PTB in a large and sociodemographically diverse sample. ABCD covers the spectrum from FTB to very PTB (≤32 weeks GA). There is a growing interest in characterizing brain differences in children born moderately preterm (>32 and <37 weeks GA), given evidence of increased cognitive and behavioral challenges relative to their FTB peers (Romeo et al., 2010, 2016; Potijk et al., 2013; Brumbaugh et al., 2016; Stene-Larsen et al., 2016). Further, there is evidence for worse outcomes in boys following PTB (Whitfield et al., 1997; Hindmarsh et al., 2000; Wood et al., 2005; Hintz et al., 2006; Young et al., 2016; Urben et al., 2017), motivating examination of sex differences (Kesler et al., 2004, 2008).

We use an estimation statistics approach (Calin-Jageman and Cumming, 2019) to map alterations in cortical thickness (CT), SA, and subcortical volumes, reporting effect sizes with confidence intervals (CIs), and considering impact of brain size controls, MRI data quality, sex differences, effects in moderate and very PTB and impact of birthweight. We hope that this comprehensive examination of associations between PTB and brain structure provides a clearer picture of long-term brain structural alterations in PTB.

## Materials and Methods

### Participants

The ABCD study is following roughly 11,500 participants from the ages of 9–11 into early adulthood at 21 sites across the United States and was designed to reflect sociodemographic diversity (Garavan et al., 2018). For our analyses, we used the baseline cohort of the ABCD Study Release 3.0, collected from children at 9–11 years of age (48% female, 52% male). The recruitment and sampling procedure of the ABCD Study were designed to be relatively sociodemographically representative of the United States population (Garavan et al., 2018).

### MRI acquisition and quality control

For the analyses described here, we looked at the structural characteristics derived from T1-weighted images. Full details on MRI image acquisition is described elsewhere (Casey et al., 2018; Hagler et al., 2019). Briefly, whole-brain T1-weighted images were collected with 1 mm isotropic voxels and varying parameters across vendors [slices: 176–256; field of view (FoV) 256 × 240–256; TE = 2–2.9 ms; flip angle 8°]. Regional measures derived from imaging were downloaded from the ABCD Data Release 3.0. Processing steps described in brief in the ABCD Release Notes include: images were corrected for gradient nonlinearity distortions (Jovicich et al., 2006), intensity nonuniformity correction was applied based on tissue segmentation and sparse spatial smoothing, images were resampled with 1 mm isotropic voxels into rigid alignment with an atlas brain. Cortical surface reconstruction was completed using FreeSurfer v5.3.0, which included skull-stripping (Ségonne et al., 2004), white matter segmentation, initial mesh creation (Dale et al., 1999), correction of topological defects (Fischl et al., 2001; Ségonne et al., 2007), surface optimization (Dale and Sereno, 1993; Dale et al., 1999; Fischl and Dale, 2000), and nonlinear registration to a spherical surface-based atlas (Fischl et al., 1999). Analyses included here used cortical thickness (Fischl and Dale, 2000) and surface area measures (Joyner et al., 2009; Chen et al., 2012) labeled using the 74 region Destrieux atlas-based classification (Destrieux et al., 2010) as well as subcortical structures labeled with atlas-based segmentation (Fischl et al., 2002), including 16 subcortical structures, cerebellar gray and white matter in each hemisphere, and six ventricular regions.

We used image quality ratings provided with the ABCD data release to filter the sample based on quality of the T1-weighted MRI images. Image quality was rated from 0 to 3 (0 = absent; 1 = mild; 2 = moderate; 3 = severe) on five components of the MRI image quality: motion, pial overestimation, white matter underestimation, inhomogeneity, and artifact. Further, a score for findings on the MRI image was reported from 0 to 4 (0 = Image artifacts prevent radiology read; 1 = No abnormal findings; 2 = Normal anatomic variant of no clinical significance; 3 = Consider clinical referral; 4 = consider immediate clinical referral). Any participant who had a score of 2 or above in any of the five components of the MRI image quality or who did not have a score of 1 or 2 in the MRI findings score were excluded. Because head motion can influence data quality and subsequent analyses (Makowski et al., 2019), we also created a more stringent subsample where all participants had a motion rating of 0 (i.e., absent). Analyses were repeated in this “stringent quality check (QC)” sample.

### Discovery and replication samples

To assess the generalizability of findings, we pseudo-randomly divided the data into “discovery” and “replication” samples all of whom passed the main MRI QC. ∼80% of the sample (*N* = 7528) was used as the discovery sample, and the remaining 20% of the sample (*N* = 2139) were used as a replication sample. The ABCD Study has 21 data collection sites and four of these sites were enriched for the sampling of twins (sites 2, 14, 19, and 20) and therefore also had higher rates of PTB as PTB is more likely in twin pregnancies (Santana et al., 2018). Thus, to ensure that we had a similar proportion of PTB children between the discovery and replication samples, we assigned three of the sites enriched for twin sampling (sites 2, 14, and 20) to the discovery sample and the remaining site enriched for twin sampling (site 19) to the replication sample. The remaining sites were pseudo-randomly assigned to samples to achieve an 80/20 ratio of participants (sites 3–10, 12, 13, 15, 17, 18, and 21 in the discovery sample and sites 1, 11, and 16 in the replication sample).

### Preterm birth analysis in the discovery sample

**Figure 1.**
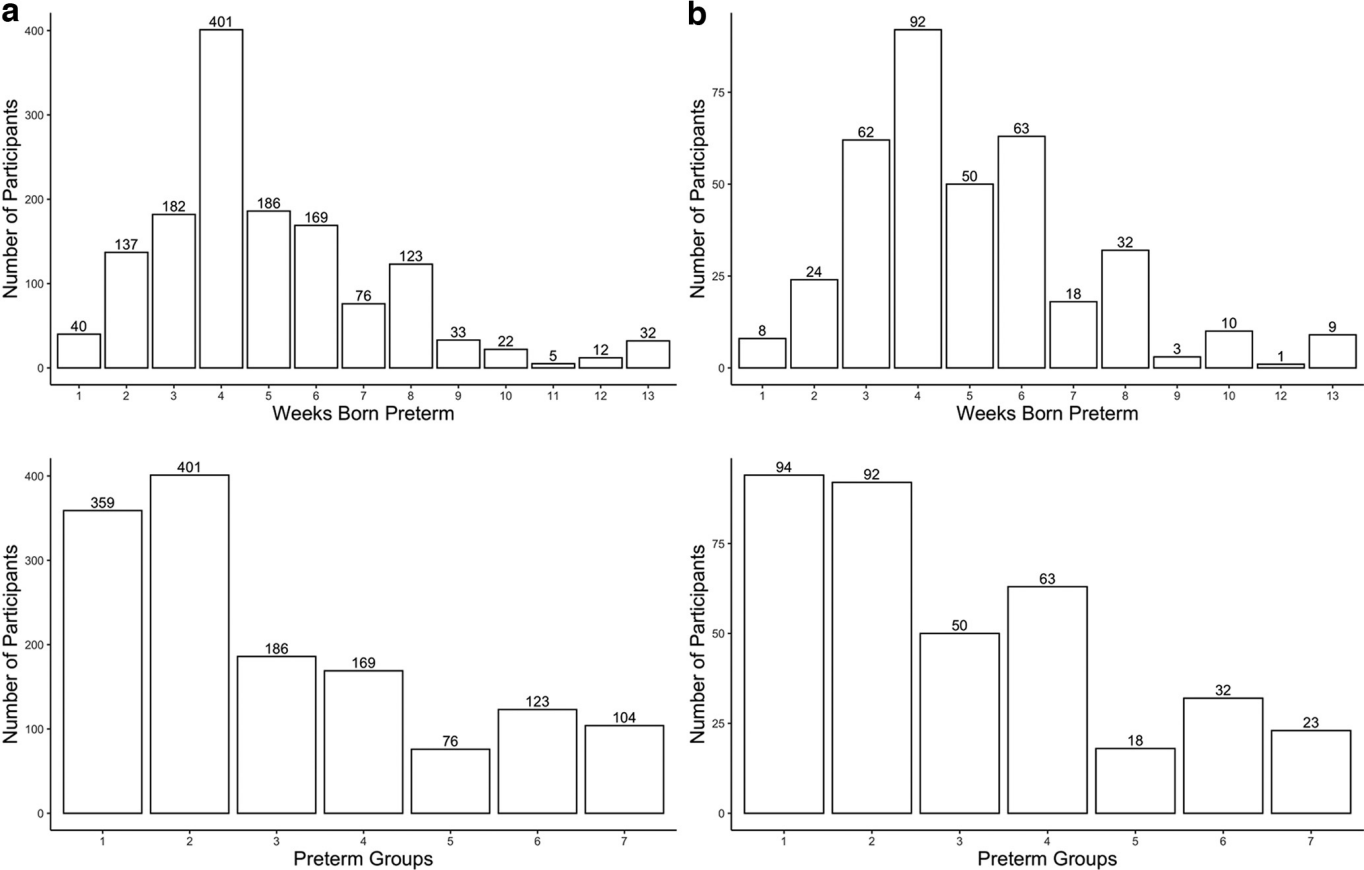
Distribution of preterm born children in the (***a***) discovery sample and (***b***) holdout sample. The top chart displays the number of participants associated with a given number of weeks born preterm. The bottom chart displays the number of participants in each preterm group. Children born at one to three weeks preterm were grouped into preterm group 1. Children born at more than eight weeks preterm were grouped into preterm group 7.

Analyses of PTB focused on two questions asked of parents: “Was the child born prematurely?” and “About how many weeks premature was the child when they were born?” Children were included in a FTB group if they responded no to the first question; for the discovery sample with liberal QC this was *N* = 6000. At most sites, ∼10% of parents reported that their child was born preterm. At four sites, where recruitment was enriched for twins, the rate of preterm birth was higher (∼40%). Given that lower GA is associated with increased risk of adverse outcomes, analyses were run assessing linear effects of weeks born preterm. Among parents who reported that their child was born preterm, smaller numbers were reported for infants born 1 (*N* = 40), 2 (*N* = 137), or 3 (*N* = 182) weeks preterm, relative to four (*N* = 401) weeks preterm (these Ns are for the discovery sample with liberal QC). After four weeks preterm there was a monotonically decreasing number of participants with increasing number of weeks preterm (Fig. 1). In the present study, we consider the children reported to be born one to three weeks preterm as being born at early term (37–39 weeks GA). These children were coded together as 1 (*N* = 359). Next, we assigned numerical values associated with an increasing number of weeks born preterm, with four weeks coded as 2 (*N* = 401), five weeks coded as 3 (*N* = 186), six weeks coded as 4 (*N* = 169), seven weeks coded as 5 (*N* = 76), and eight weeks coded as 6 (*N* = 123). As the number of children in each incremental week became increasingly smaller, children born nine or more weeks preterm were coded as 7 (*N* = 104; Fig. 1). FTB children (*N* = 6000) were coded as 0. Follow-up analyses were conducted to assess the relative contribution of moderate PTB (four to seven weeks preterm, i.e., 33–36 weeks GA; Blencowe et al., 2012), or very PTB (more than or equal to eight weeks preterm, i.e., ≤32 weeks GA) to alterations in brain morphology compared with FTB. These analyses were conducted by assigning FTB as 0, moderate PTB as 1 and excluding very PTB participants in the moderate PTB analyses relative to FTB, and with FTB assigned 0, very PTB assigned 1 and moderate PTB excluded in the very PTB analyses relative to FTB.

**Figure 2.**
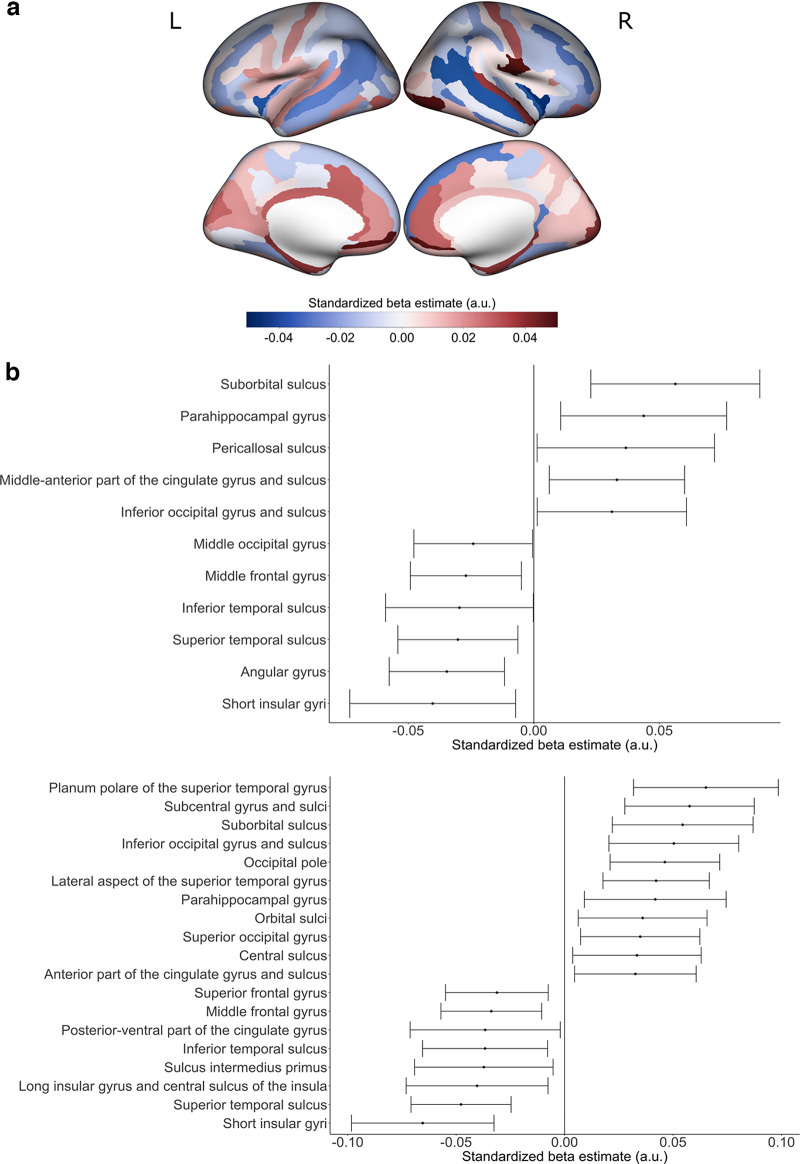
Preterm birth associations with cortical thickness with a linear control for mean hemispheric cortical thickness. ***a***, Positive β estimates are shown in red, indicating thicker cortical thickness with shorter gestational age. Negative β estimates are shown in blue, indicating thinner cortical thickness with shorter gestational age. ***b***, Estimated standardized βs of cortical regions whose 99% confidence interval do not overlap 0 are displayed for (top) left hemisphere cortical thickness and (bottom) right hemisphere cortical thickness. For the estimated standardized βs with their 99% confidence intervals for all cortical regions, refer to Extended Data Figure 2-1. For preterm birth associations with cortical thickness without a control for mean hemispheric cortical thickness, refer to Extended Data Figure 2-2. For preterm birth associations with cortical thickness in the stringent quality subsample, refer to Extended Data Figure 2-3. For birth complication associations with cortical thickness, refer to Extended Data Figure 2-4. For preterm birth associations with cortical thickness with a linear control for birth complications, refer to Extended Data Figure 2-5. For a replication of Figure 2 with ComBat used as the method for controlling variation between sites, refer to Extended Data Figure 2-6. a.u. = arbitrary units. L = left hemisphere; R = right hemisphere.

10.1523/ENEURO.0196-22.2023.f2-1 Extended Data Figure 2-1 Preterm birth associations with cortical thickness with a linear control for mean hemispheric cortical thickness in all cortical regions. Estimated standardized βs are displayed with their 99% confidence intervals separately for the (top) left hemisphere cortical thickness and the (bottom) right hemisphere cortical thickness. a.u. = arbitrary units. Download Figure 2-1, TIF file.

10.1523/ENEURO.0196-22.2023.f2-2 Extended Data Figure 2-2 Preterm birth associations with cortical thickness without a control for mean hemispheric cortical thickness. ***a***, Positive β estimates are shown in red, indicating thicker cortical thickness with shorter gestational age. Negative β estimates are shown in blue, indicating thinner cortical thickness with shorter gestational age. ***b***, Estimated standardized βs of cortical regions whose 99% confidence interval do not overlap 0 are displayed for (top) left hemisphere cortical thickness and (bottom) right hemisphere cortical thickness. a.u. = arbitrary units. Download Figure 2-2, TIF file.

**Figure 3.**
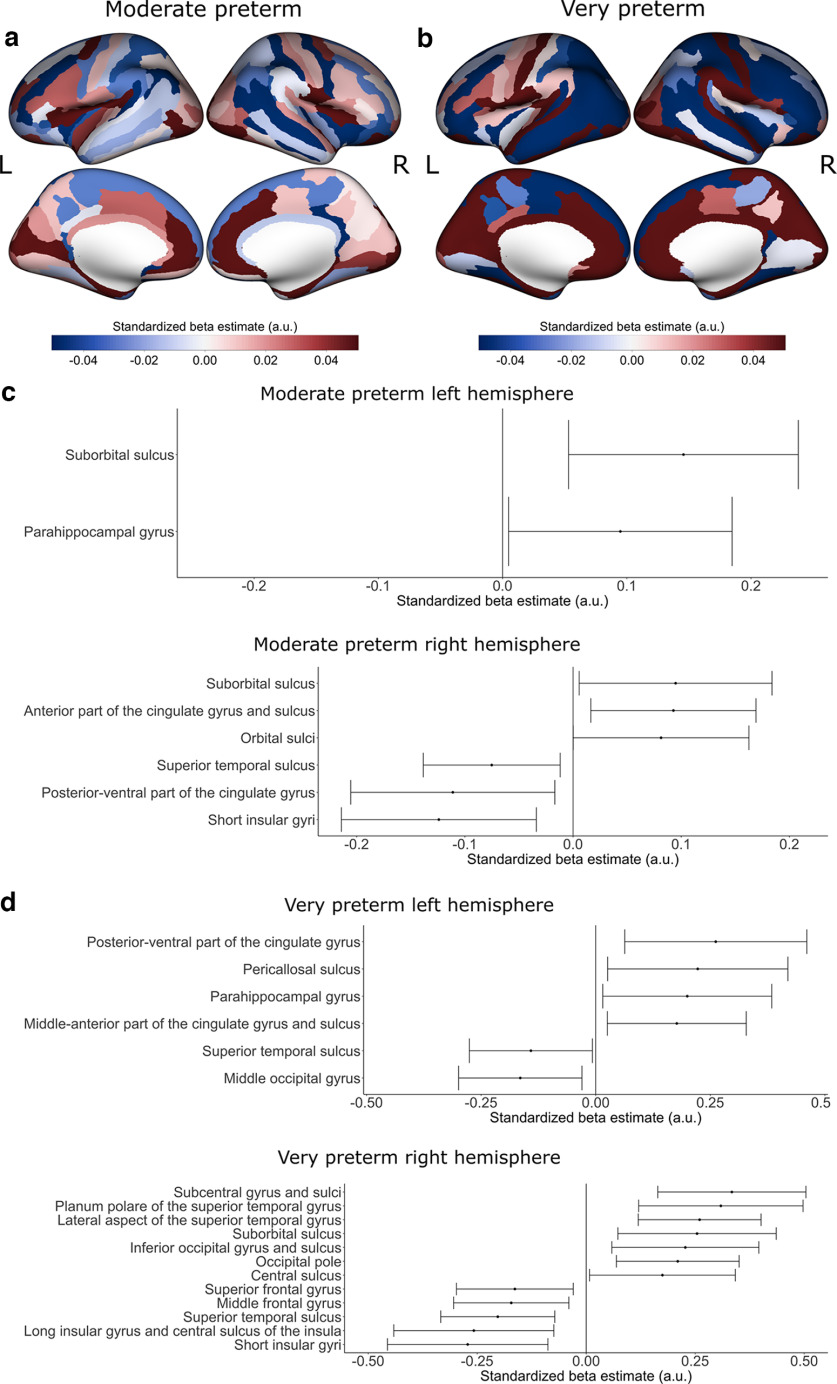
Preterm birth associations with cortical thickness comparing moderate preterm birth relative to full term birth and very preterm birth relative to full term birth. Positive β estimates are shown in red, indicating thicker cortical thickness with shorter gestational age, and negative β estimates are shown in blue, indicating thinner cortical thickness with shorter gestational age, for (***a***) moderate preterm birth relative to full term birth and (***b***) very preterm birth relative to full term birth. Estimated standardized βs of cortical regions whose 99% confidence interval do not overlap 0 are displayed for (top) left hemisphere cortical thickness and (bottom) right hemisphere cortical thickness for (***c***) moderate preterm birth relative to full term birth and (***d***) very preterm birth relative to full term birth. Patterns of effects were generally similar between groups, although with several regions showing larger effect size estimates for the very preterm birth sample. For the estimated standardized βs with their 99% confidence intervals for all cortical regions, refer to Extended Data Figure 3-1. a.u. = arbitrary units. L = left hemisphere; R = right hemisphere.

10.1523/ENEURO.0196-22.2023.f2-3 Extended Data Figure 2-3 Preterm birth associations with cortical thickness in stringent quality subsample. ***a***, Positive β estimates are shown in red, indicating thicker cortical thickness with shorter gestational age. Negative β estimates are shown in blue, indicating thinner cortical thickness with shorter gestational age. ***b***, Estimated standardized βs of cortical regions whose 99% confidence interval do not overlap 0 are displayed for (top) left hemisphere cortical thickness and (bottom) right hemisphere cortical thickness. a.u. = arbitrary units. Download Figure 2-3, TIF file.

**Figure 4.**
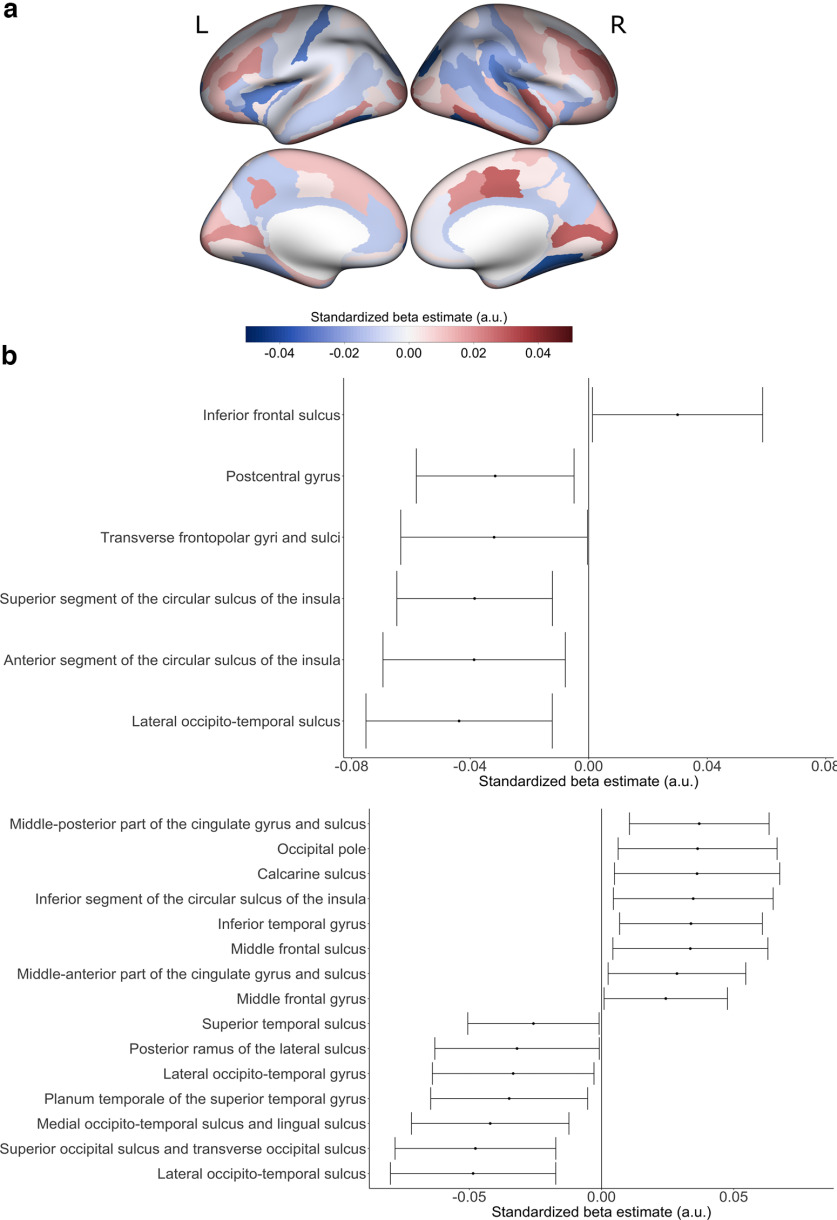
Preterm birth associations with cortical surface area with a linear control for total hemispheric cortical surface area. ***a***, Positive β estimates are shown in red, indicating greater surface area with shorter gestational age. Negative β estimates are shown in blue, indicating smaller surface area with shorter gestational age. ***b***, Estimated standardized βs of cortical regions whose 99% confidence interval do not overlap 0 are displayed for (top) left hemisphere surface area and (bottom) right hemisphere surface area. For the estimated standardized βs with their 99% confidence intervals for all cortical regions, refer to Extended Data Figure 4-1. For preterm birth associations with cortical surface area without a control for total hemispheric cortical surface area, refer to Extended Data Figure 4-2. For preterm birth associations with cortical surface area in the stringent quality subsample, refer to Extended Data Figure 4-3. For preterm birth associations with cortical surface area in moderate preterm birth relative to full term birth and very preterm birth relative to full term birth, refer to Extended Data Figure 4-4. For birth complication associations with cortical surface area, refer to Extended Data Figure 4–5. For preterm birth associations with cortical surface area with a linear control for birth complications, refer to Extended Data Figure 4-6. a.u. = arbitrary units. L = left hemisphere; R = right hemisphere.

10.1523/ENEURO.0196-22.2023.f2-4 Extended Data Figure 2-4 Birth complication associations with cortical thickness. ***a***, Positive β estimates are shown in red, indicating thicker cortical thickness in children who experienced a birth complication requiring hospital stay. Negative β estimates are shown in blue, indicating thinner cortical thickness in children who experienced a birth complication requiring hospital stay. ***b***, Estimated standardized βs of cortical regions whose 99% confidence interval do not overlap 0 are displayed for right hemisphere cortical thickness; all estimated standardized βs of cortical regions in the left hemisphere had a 99% confidence interval that overlapped 0. a.u. = arbitrary units. Download Figure 2-4, TIF file.

**Figure 5.**
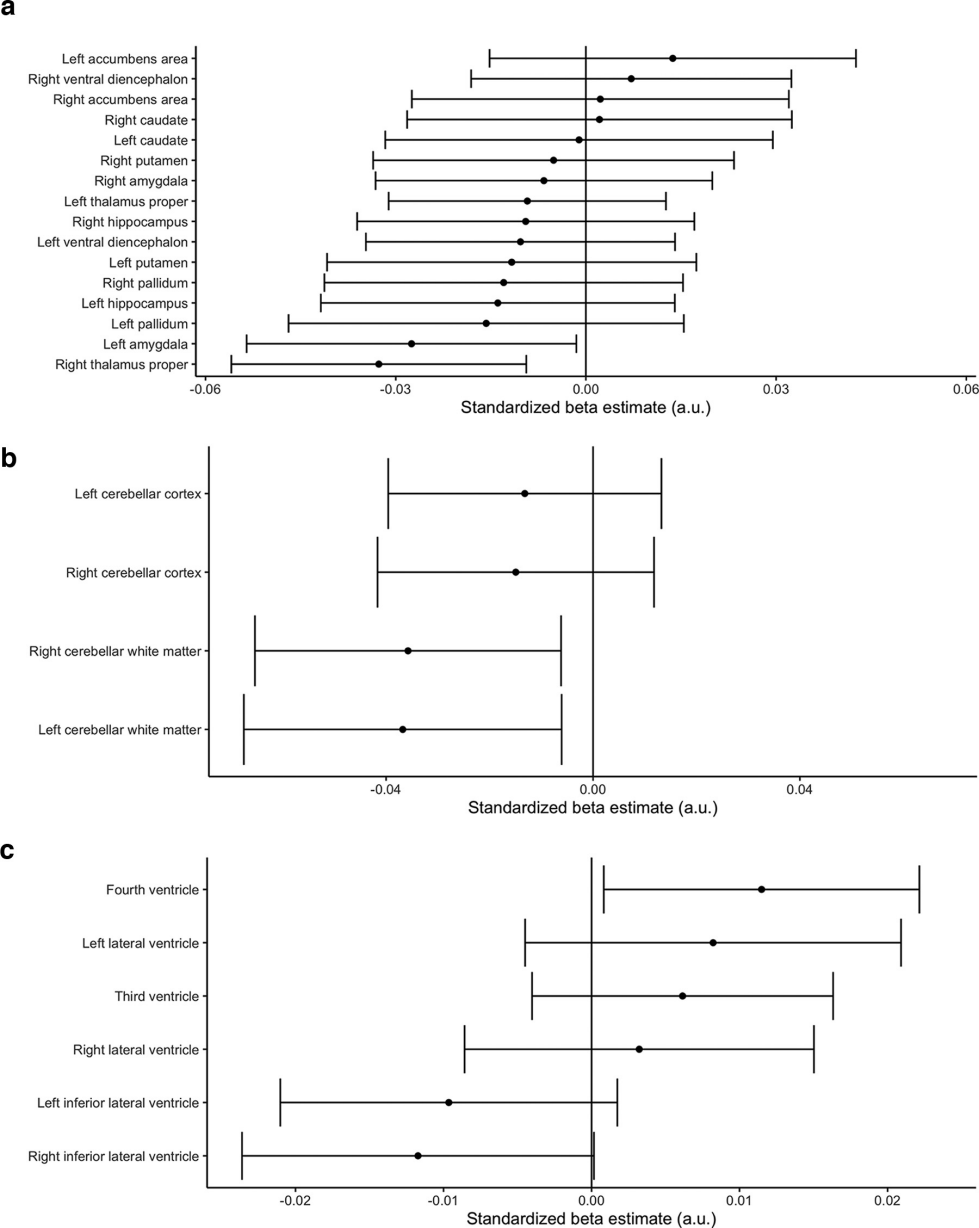
Preterm birth associations with (***a***) subcortical, (***b***) cerebellar, and (***c***) log-transformed ventricular volumes with a linear control for total brain volume. Estimated standardized βs with 99% confidence intervals are shown. Only the volumes of right thalamus, left amygdala, bilateral cerebellar white matter, and log-transformed fourth ventricle had high confidence of non-zero effects. For preterm birth associations with subcortical, cerebellar, and log-transformed ventricular volumes without a control for total brain volume, refer to Extended Data Figure 5-1. For preterm birth associations with subcortical, cerebellar, and log-transformed ventricular volumes in the stringent quality subsample, refer to Extended Data Figure 5-2. For preterm birth associations with subcortical, cerebellar, and log-transformed ventricular volumes in moderate preterm birth relative to full term birth, refer to Extended Data Figure 5-3. For preterm birth associations with subcortical, cerebellar, and log-transformed ventricular volumes in very preterm birth relative to full term birth, refer to Extended Data Figure 5-4. For birth complication associations with subcortical, cerebellar, and log-transformed ventricular volumes, refer to Extended Data Figure 5-5. For preterm birth associations with subcortical, cerebellar, and log-transformed ventricular volumes with a linear control for birth complications, refer to Extended Data Figure 5-6. a.u. = arbitrary units.

10.1523/ENEURO.0196-22.2023.f2-5 Extended Data Figure 2-5 Preterm birth associations with cortical thickness with a linear control for birth complications. ***a***, Positive β estimates are shown in red, indicating thicker cortical thickness with shorter gestational age. Negative β estimates are shown in blue, indicating thinner cortical thickness with shorter gestational age. ***b***, Estimated standardized βs of cortical regions whose 99% confidence interval do not overlap 0 are displayed for (top) left hemisphere cortical thickness and (bottom) right hemisphere cortical thickness. a.u. = arbitrary units. Download Figure 2-5, TIF file.

**Figure 6.**
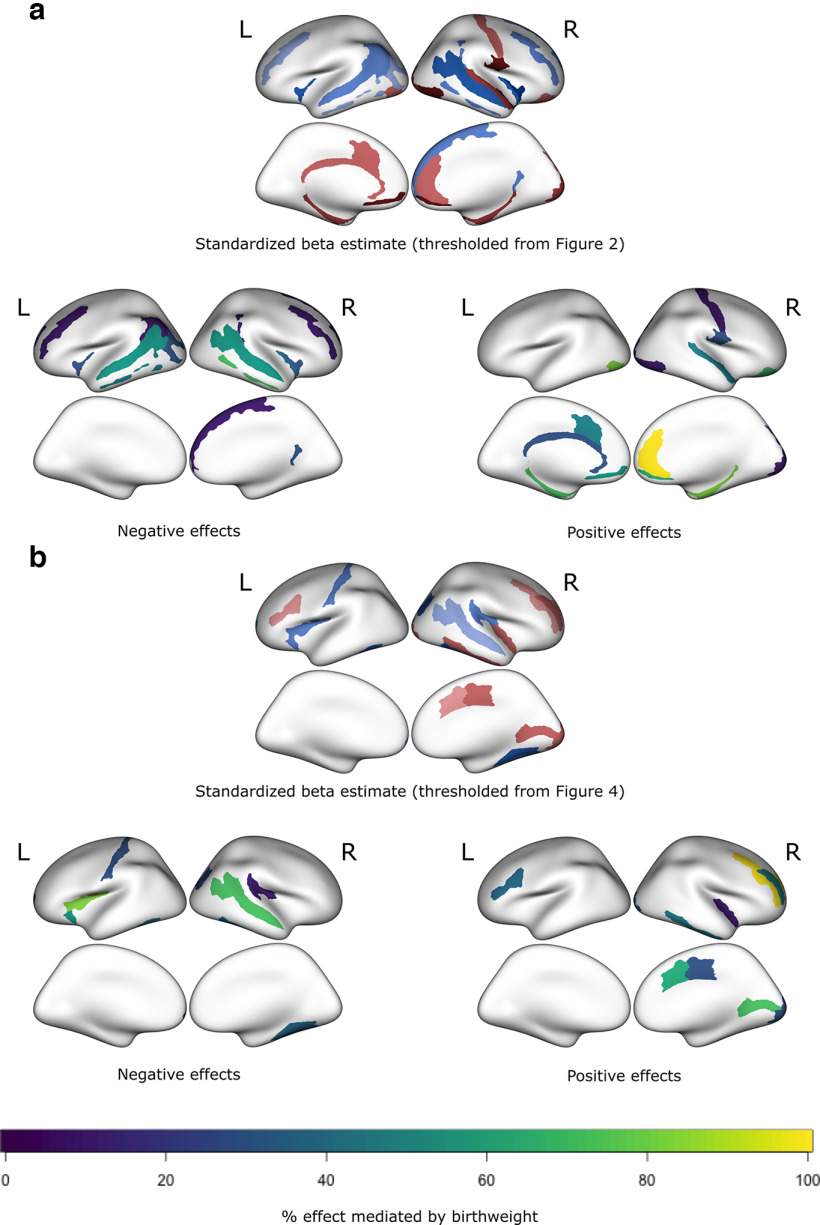
Potential mediating effect of birthweight on (***a***) cortical thickness and (***b***) cortical surface area. High confidence estimated effects are indicated in the upper-middle of each panel, gathered from Figures 2 and 4, with negative effects in blue-scale and positive effects in red-scale. For visualization of effects, if inclusion of birthweight led to a sign change in the effect of preterm birth on a brain region, we indicated this as a 100% mediation by birthweight and if the effect of preterm birth increased after accounting for birthweight, we indicated this as a 0% mediation by birthweight. Most regions showed attenuation of parameter estimates related to preterm birth when birthweight was included in the models, suggesting partial mediation effects. Regions in yellow show full attenuation of preterm birth effects by birthweight (i.e., 0% of the preterm birth effects were retained after accounting for birthweight); regions in purple indicate no attenuation of preterm birth effects by birthweight (i.e., 100% of the preterm birth effects were retained after accounting for birthweight). L = left hemisphere; R = right hemisphere.

10.1523/ENEURO.0196-22.2023.f2-6 Extended Data Figure 2-6 Preterm birth associations with cortical thickness with a linear control for mean hemispheric cortical thickness using ComBat as the method of controlling variation between sites. ***a***, Positive β estimates are shown in red, indicating thicker cortical thickness with shorter gestational age. Negative β estimates are shown in blue, indicating thinner cortical thickness with shorter gestational age. ***b***, Estimated standardized βs of cortical regions whose 99% confidence interval do not overlap 0 are displayed for (top) left hemisphere cortical thickness and (bottom) right hemisphere cortical thickness. a.u. = arbitrary units. Download Figure 2-6, TIF file.

### Statistical analyses and inferences

Statistical analyses were conducted using R version 3.6.0 (R Core Team, 2021) on a PC computer running Windows 10. Linear mixed models in the gamm4 package were used to quantify linear associations between gestational age, i.e., weeks born preterm, and regional brain structure. R code was adapted from analysis scripts within the Data Exploration and Analysis Portal provided by the ABCD study (ABCD Study). The dependent variables of interest included cortical thickness, cortical surface area, subcortical gray matter volumes, cerebellar volumes, and ventricular volumes. The following fixed-effects covariates were included in all regression models: age, sex, race/ethnicity, household income, highest parental education level, and a binary variable indicating whether the participant was a singleton birth or a multiple birth. We also conducted a set of analysis in which we controlled for birth complications. The birth complications variable was a binary variable asking parents if their child had any birth complications requiring hospital stay for at least one month. We report both associations with birth complications and effects of preterm birth when this covariate is included. The random-effects covariates included in each of the regression models were the scanner ID to control for site and scanner effects, and family ID to control for sibling status. All continuous outcome and predictor variables were standardized to obtain standardized β coefficients. The remaining variables (race/ethnicity, household income, highest parental education level, and whether a participant had a twin) were treated as categorical variables.

As regional metrics scale with overall brain size, and overall brain size is associated with preterm group (standardized β: −0.03, SD: 0.01) we used two approaches to model the data: (1) absolute effects uncontrolled for brain size, and (2) relative effect models controlling for linear associations with brain size. In models using brain size controls, we used a parameter appropriate to the structural parameter. For cortical thickness models, we used mean hemispheric cortical thickness and, for cortical surface area models, we used total hemispheric surface area. For subcortical volume models (subcortical gray matter volumes, cerebellar volumes, and ventricular volumes), we used whole brain volume.

To assess model fit, we considered the distribution of the residuals by analyzing the quantile-quantile plot (Q-Q plot) for three randomly selected regions. All of the residuals examined had a linear Q-Q plot indicating normality except for those associated with the ventricular models, whose Q-Q plot was exponentially curved. We therefore used log-transformed regional ventricle volumes as the dependent variables in the ventricular models, which produced linear Q-Q plots of the residual distribution.

In line with an estimation statistics framework (Calin-Jageman and Cumming, 2019), results are presented using standardized β coefficients as effect size estimates with 99% confidence intervals. Confidence intervals were set at 99% rather than 95% because of the large sample size and multiple tests performed in parallel. We examined patterns across regions and regions where effects were of high confidence (99% confidence intervals not including zero). Positive effects (i.e., larger in PTB) are shown in shades of red, while negative effects (i.e., smaller in PTB) are in shades of blue in the figures to follow.

### Considering mediation by birthweight

PTB is a complex process that increases risk for perinatal injury as well as lower birthweight relative to term-born peers. As being born earlier is associated with lower birthweight (in this sample, birthweight was associated with weeks born preterm at standardized β = −0.299170, *p* < 2e-16), and birthweight has been associated with variation in brain structure in childhood (Walhovd et al., 2012), we assessed whether including birthweight as a covariate in PTB models would reduce associations between PTB and brain structure, thereby suggesting a potential mediating effect. In brain regions where PTB had a high confidence association with brain structure (i.e., in which 99% confidence intervals for β coefficients in the brain size-controlled models did not overlap 0), we assessed how including birthweight as a covariate reduced effect size estimates (calculated as a percentage of attenuation of the original effect of preterm without birthweight as a covariate).

### Sex differences in PTB associations with brain structure

We considered sex differences in associations between PTB and brain structure by (1) conducting analyses for girls and boys separately, and (2) adding a sex*PTB interaction term to models of the full sample.

### Assessing generalizability from the discovery sample to the replication sample

We assessed generalizability of findings from the discovery to the replication sample by using the inverse β weights for each brain region to predict GA (i.e., how many weeks born preterm). Specifically, in models that were originally built as ROI_value ∼ β*PTB_category, for each high confidence region (99% CIs not overlapping zero) in the discovery sample, the effect estimates were inverted and multiplied by the ROI_value to predict GA in the replication sample and these predictions were averaged across regions of interest (ROIs). Each parameter was assessed separately (CT, SA, subcortical, cerebellar, and ventricular regions) and a model was constructed considering all parameters. Predictive accuracy was assessed using a Spearman correlation because modeled/predicted values were categorical rather than continuous and confidence intervals around predictions were obtained with bootstrap resampling.

### Code accessibility

The code described in the paper is freely available online at https://github.com/BrayNeuroimagingLab/BNL_open/tree/main/abcdPTB. The code is available as Extended Data 1.

## Results

### Characteristics of ABCD sample in relation to preterm birth

In the discovery sample, after exclusions because of poor data quality and missing demographic data, our sample included 6000 FTB children, and 1418 PTB children. Demographics and current characteristics are presented in which mean differences and 99% confidence intervals are shown for continuous variables and *p*-values for Fisher’s exact tests are shown for categorical variables (Table 1). Children born preterm were on average ∼1.5 months older in unadjusted age (i.e., not corrected for preterm weeks) when they were imaged compared with the FTB children and the ratio of boys to girls was slightly higher in the PTB group. Both FTB and PTB group included sociodemographically and ethnically diverse participants. As expected, in terms of perinatal characteristics, children born preterm had lower birthweight and were more likely to be part of a multiple birth. Children in the FTB and PTB groups did not differ in height at the time of recruitment into ABCD, suggesting a catch-up in physical growth. General cognitive ability as quantified by the NIH Toolbox Total Composite score (Akshoomoff et al., 2013) was higher in the FTB group.

**Table 1:** Main sample demographics

			Preterm group			
		Level	Full term	Preterm	Mean difference (99% CI)	Fisher’s exact *p*-value
	*N*		6000	1418		
Demographics	Age [unadjusted months; mean (SD)]		118.91 (7.47)	120.38 (7.35)	−1.47 (−2.03, −0.91)	
	Sex (%)	Female	2943 (49.1)	659 (46.5)		0.08
		Male	3057 (51.0)	759 (53.5)		
	Race/ethnicity (%)	White	2984 (49.7)	798 (56.3)		<0.001
		Hispanic	1308 (21.8)	281 (19.8)		
		Black	954 (15.9)	159 (11.2)		
		Asian	135 (2.3)	16 (1.1)		
		Other	618 (10.3)	163 (11.5)		
	Household income bracket (%)	<$50K	1625 (29.7)	333 (25.3)		0.003
		$50K–<$100K	1477 (27.0)	371 (28.2)		
		≥100K	2375 (43.4)	612 (46.5)		
	Highest parental education (%)	<HS	299 (5.0)	43 (3.0)		<0.001
		HS Diploma/GED	558 (9.3)	123 (8.7)		
		Some College	1518 (25.3)	394 (27.8)		
		Bachelor	1449 (24.2)	407 (28.7)		
		Postgraduate	2168 (36.2)	450 (31.8)		
Perinatal characteristics	Birth weight [oz, mean (SD)]		118.53 (19.17)	85.60 (22.08)	32.93 (31.27, 34.60)	
	Twin status (%)	No	5367 (89.5)	601 (42.4)		<0.001
		Yes	628 (10.5)	816 (57.6)		
Current characteristics	Height [inches, mean (SD)]		55.32 (3.31)	55.45 (3.19)	−0.12 (−0.37, 0.12)	
	NIH Toolbox Total Composite T-Score [mean (SD)]		48.27 (11.26)	46.70 (10.96)	1.57 (0.69, 2.44)	

For continuous variables, mean difference and 99% confidence intervals are shown; for categorical variables, *p*-values for Fisher’s exact test are shown. We note that because of the large sample size in the Adolescent Brain Cognitive Development Study, small differences can be statistically significant. Demographic variables (age, sex, race/ethnicity, household income, and highest parental education) and twin status were included as covariates in the analyses of the effects of preterm birth on brain structure. CI = confidence interval; SD = standard deviation.

### Effects of preterm birth on brain structure

#### Cortical thickness (CT)

In models assessing linear associations of CT with weeks born preterm and controlling for mean hemispheric CT (Fig. 2; Extended Data Fig. 2-1), we found an overall pattern of cortical thinning in temporoparietal and dorsal prefrontal regions and thickening of medial orbitofrontal and occipital regions with increasing weeks born preterm. Regions with high confidence of cortical thinning (i.e., 99% confidence intervals do not include zero) included the left angular gyrus, bilateral superior and inferior temporal sulci as well as the bilateral middle frontal gyrus, bilateral short insular gyrus, right long insular gyrus, and central sulcus of the insula and right sulcus intermedius primus. Regions with high confidence of cortical thickening with increasing weeks preterm include orbitofrontal (bilateral suborbital sulcus, right orbital sulcus), occipital (right occipital pole, right superior occipital gyrus, bilateral inferior occipital gyrus and sulcus), and cingulate (left mid anterior gyrus and sulcus, left pericallosal sulcus) regions as well as bilateral parahippocampal gyrus, and right central sulcus and subcentral gyrus and sulci.

CT models uncontrolled for mean hemispheric CT had similar negative effects but less sensitivity to positive effects (Extended Data Fig. 2-2). Analyses were repeated in the stringent MRI QC sample and the pattern of results was similar, though, because of a smaller sample size, confidence intervals were wider. Also, we note that several effect estimates were larger in the stringent sample, suggesting some attenuation related to measurement error in the liberal sample (Extended Data Fig. 2-3).

We considered the relative role of moderate and very PTB in driving these effects. We found that effects for moderate and very PTB followed a similar pattern as the continuous preterm weeks model, but effect sizes were larger in several regions in the analysis that examined very PTB (Fig. 3).

10.1523/ENEURO.0196-22.2023.f3-1 Extended Data Figure 3-1 Preterm birth associations with cortical thickness comparing (***a***) moderate preterm birth relative to full term birth and (***b***) very preterm birth relative to full term birth in all cortical regions. Estimated standardized βs are displayed with their 99% confidence intervals separately for the (top) left hemisphere cortical thickness and the (bottom) right hemisphere cortical thickness. a.u. = arbitrary units. Download Figure 3-1, TIF file.

#### Surface area (SA)

In models assessing linear associations of SA with weeks born preterm and controlling for total hemispheric SA, effects of preterm weeks paralleled those seen in the CT models in temporal and occipital regions but diverged in lateral prefrontal and ventral visual regions (Fig. 4; Extended Data Fig. 4-1). Regions with high confidence of reduced SA with increasing weeks born preterm include bilateral lateral occipito-temporal sulcus, left superior and anterior circular sulcus of the insula, left postcentral gyrus, left transverse frontopolar gyri and sulci, right planum temporale of superior temporal gyrus, right posterior ramus of the lateral sulcus, right lateral occipito-temporal gyrus and right superior occipital and transverse occipital sulcus. Regions with high confidence of increased SA with increasing weeks born preterm include right middle frontal gyrus and sulcus, left inferior frontal sulcus, right middle-anterior and middle-posterior cingulate gyrus and sulcus, right inferior temporal gyrus, right inferior segment of the circular sulcus of the insula, right calcarine sulcus and occipital pole. In models uncontrolled for total hemispheric SA, there was a greater sensitivity to negative effects and complete attenuation of positive effects (Extended Data Fig. 4-2). In the stringent QC sample (Extended Data Fig. 4-3), confidence intervals were wider as expected because of lower sample size. The only positive effect that maintained high confidence (i.e., where the 99% confidence interval did not include zero) in the stringent QC sample was the right inferior segment of the circular sulcus of the insula. A large number of regions showing high confidence of both positive and negative effects of PTB on SA in the right hemisphere were largely attenuated in stringent QC sample. Extended Data Figure 4-4 shows that the pattern of effects was similar when considering moderate PTB or very PTB in relation to FTB, although most of the effects largely failed to achieve high confidence (i.e., their 99% confidence interval included 0).

10.1523/ENEURO.0196-22.2023.f4-1 Extended Data Figure 4-1 Preterm birth associations with cortical surface area with a linear control for total hemispheric cortical surface area in all cortical regions. Estimated standardized βs are displayed with their 99% confidence intervals separately for the (top) left hemisphere surface area and the (bottom) right hemisphere surface area. a.u. = arbitrary units. Download Figure 4-1, TIF file.

10.1523/ENEURO.0196-22.2023.f4-2 Extended Data Figure 4-2 Preterm birth associations with cortical surface area without a control for total hemispheric cortical surface area. ***a***, Positive β estimates are shown in red, indicating greater surface area with shorter gestational age. Negative β estimates are shown in blue, indicating smaller surface area with shorter gestational age. ***b***, Estimated standardized βs of cortical regions whose 99% confidence interval do not overlap 0 are displayed for (top) left hemisphere surface area and (bottom) right hemisphere surface area. a.u. = arbitrary units. Download Figure 4-2, TIF file.

10.1523/ENEURO.0196-22.2023.f4-3 Extended Data Figure 4-3 Preterm birth associations with cortical surface area in stringent quality subsample. ***a***, Positive β estimates are shown in red, indicating greater surface area with shorter gestational age. Negative β estimates are shown in blue, indicating smaller surface area with shorter gestational age. ***b***, Estimated standardized βs of cortical regions whose 99% confidence interval do not overlap 0 are displayed for (top) left hemisphere surface area and (bottom) right hemisphere surface area. a.u. = arbitrary units. Download Figure 4-3, TIF file.

10.1523/ENEURO.0196-22.2023.f4-4 Extended Data Figure 4-4 Preterm birth associations with cortical surface area in moderate preterm birth relative to full term birth and very preterm birth relative to full term birth. Positive β estimates are shown in red, indicating greater surface area with shorter gestational age, and negative β estimates are shown in blue, indicating smaller surface area with shorter gestational age, for (***a***) moderate preterm birth relative to full term birth and (***b***) very preterm birth relative to full term birth. Estimated standardized βs of cortical regions whose 99% confidence interval do not overlap 0 are displayed for (top) left hemisphere surface area and (bottom) right hemisphere surface area for (***c***) moderate preterm birth relative to full term birth and (***d***) very preterm birth relative to full term birth. a.u. = arbitrary units. Download Figure 4-4, TIF file.

10.1523/ENEURO.0196-22.2023.f4-5 Extended Data Figure 4-5 Birth complication associations with cortical surface area. ***a***, Positive β estimates are shown in red, indicating greater cortical surface area in children who experienced a birth complication requiring hospital stay. Negative β estimates are shown in blue, indicating smaller cortical surface area in children who experienced a birth complication requiring hospital stay. ***b***, Estimated standardized βs of cortical regions whose 99% confidence interval do not overlap 0 are displayed for right hemisphere cortical surface area; all estimated standardized βs of cortical regions in the left hemisphere had a 99% confidence interval that overlapped 0. a.u. = arbitrary units. Download Figure 4-5, TIF file.

10.1523/ENEURO.0196-22.2023.f4-6 Extended Data Figure 4-6 Preterm birth associations with cortical surface area with a linear control for birth complications. ***a***, Positive β estimates are shown in red, indicating greater cortical surface area with shorter gestational age. Negative β estimates are shown in blue, indicating smaller cortical surface area with shorter gestational age. ***b***, Estimated standardized βs of cortical regions whose 99% confidence interval do not overlap 0 are displayed for (top) left hemisphere cortical surface area and (bottom) right hemisphere cortical surface area. a.u. = arbitrary units. Download Figure 4-6, TIF file.

### Subcortical, cerebellar, and ventricular volumes

In models assessing linear effects of weeks born preterm on subcortical, cerebellar, and log-transformed ventricular volumes and controlling for total brain volume (Fig. 5), we found that the right thalamus, left amygdala and bilateral cerebellar white matter had high confidence of reduced volume with increasing weeks born preterm. The fourth ventricle had high confidence of enlarged log-transformed volume with increasing weeks born preterm. In models that did not control for total brain volume, more regions had high confidence of reduced volume with PTB (99% CIs not including zero) including bilateral thalamus, left hippocampus and ventral diencephalon, and the log-transformed right inferior lateral ventricle (Extended Data Fig. 5-1). In the stringent QC sample (Extended Data Fig. 5-2), the only region that had a high confidence of reduced volume was the right thalamus. Considering moderate PTB and very PTB group analyses (Extended Data Figs. 5-3, 5-4, respectively), each relative to FTB, some estimated effects were larger in the very PTB analysis, including log-transformed ventricle enlargement and left thalamus reduction, although, as was the case in the whole sample analyses, few effects were of high confidence.

10.1523/ENEURO.0196-22.2023.f5-1 Extended Data Figure 5-1 Preterm birth associations with (***a***) subcortical, (***b***) cerebellar, and (***c***) log-transformed ventricular volumes without a control for total brain volume. Estimated standardized βs with 99% confidence intervals are shown. a.u. = arbitrary units. Download Figure 5-1, TIF file.

10.1523/ENEURO.0196-22.2023.f5-2 Extended Data Figure 5-2 Preterm birth associations with (***a***) subcortical, (***b***) cerebellar, and (***c***) log-transformed ventricular volumes in stringent quality subsample. Estimated standardized βs with 99% confidence intervals are shown. a.u. = arbitrary units. Download Figure 5-2, TIF file.

10.1523/ENEURO.0196-22.2023.f5-3 Extended Data Figure 5-3 Preterm birth associations with (***a***) subcortical, (***b***) cerebellar, and (***c***) log-transformed ventricular volumes in moderate preterm birth relative to full term birth. Estimated standardized βs with 99% confidence intervals are shown. a.u. = arbitrary units. Download Figure 5-3, TIF file.

10.1523/ENEURO.0196-22.2023.f5-4 Extended Data Figure 5-4 Preterm birth associations with (***a***) subcortical, (***b***) cerebellar, and (***c***) log-transformed ventricular volumes in very preterm birth relative to full term birth. Estimated standardized βs with 99% confidence intervals are shown. a.u. = arbitrary units. Download Figure 5-4, TIF file.

10.1523/ENEURO.0196-22.2023.f5-5 Extended Data Figure 5-5 Birth complication associations with (***a***) subcortical, (***b***) cerebellar, and (***c***) log-transformed ventricular volumes. Estimated standardized βs with 99% confidence intervals are shown. a.u. = arbitrary units. Download Figure 5-5, TIF file.

10.1523/ENEURO.0196-22.2023.f5-6 Extended Data Figure 5-6 Preterm birth associations with (***a***) subcortical, (***b***) cerebellar, and (***c***) log-transformed ventricular volumes with a linear control for birth complications. Estimated standardized βs with 99% confidence intervals are shown. a.u. = arbitrary units. Download Figure 5-6, TIF file.

### Effects of birth complications on brain structure

When looking at the associations between birth complications and brain structure, few regions had high confidence associations. For cortical thickness (Extended Data Fig. 2-4), we saw a high confidence of increased cortical thickness in the right marginal branch of the cingulate gyrus and decreased cortical thickness in the right postcentral gyrus related to birth complications. For surface area (Extended Data Fig. 4-5), we saw increased surface area in the right middle frontal gyrus and decreased surface area in the right planum polare of the superior temporal gyrus, subcentral gyrus and sulci, and the straight gyrus. No subcortical regions had high confidence associations (Extended Data Fig. 5-5).

Next, we considered whether associations between GA and brain structure were altered when including birth complications as a linear control. For surface area (Extended Data Fig. 4-6), in the left hemisphere, the orbital sulci showed a high confidence of increased cortical surface area with decreased GA that was not seen in the primary analysis, and the decreased surface area seen in the postcentral gyrus in the primary analysis lost its high confidence when controlling for birth complications. In the right hemisphere, a lot of high confidence associations with GA were attenuated when controlling for birth complications. The regions that retained high confidence in the right hemisphere were the inferior segment of the circular sulcus of the insula, the middle-posterior part of the cingulate gyrus and sulcus, the occipital pole, the middle frontal sulcus, the inferior temporal gyrus, the medial occipito-temporal sulcus and lingual sulcus, the lateral occipito-temporal sulcus, and the superior occipital sulcus and transverse occipital sulcus.

With regards to cortical thickness (Extended Data Fig. 2-5), and subcortical, cerebellar, and log-transformed ventricular volumes (Extended Data Fig. 5-6), we did not see any appreciable changes to the results from the analyses that did not include birth complication as a covariate.

### Does birthweight mediate relationships between preterm birth and brain structure?

Figure 6 shows that for most CT and SA regions that had a high confidence of associations with PTB, effects may be partly attributed to birthweight, that is, effect sizes for PTB are reduced when birthweight is included in models (yellow indicates that effect of PTB is fully attenuated after accounting for birthweight suggesting 100% mediation by birthweight, and purple indicates that effect of PTB is fully retained after accounting for birthweight suggesting 0% mediation by birthweight). Two findings which are completely mediated by birthweight: the positive association between increasing weeks preterm and the right anterior cingulate gyrus and sulcus CT and the right middle frontal gyrus SA.

### Differences by sex

**Figure 7.**
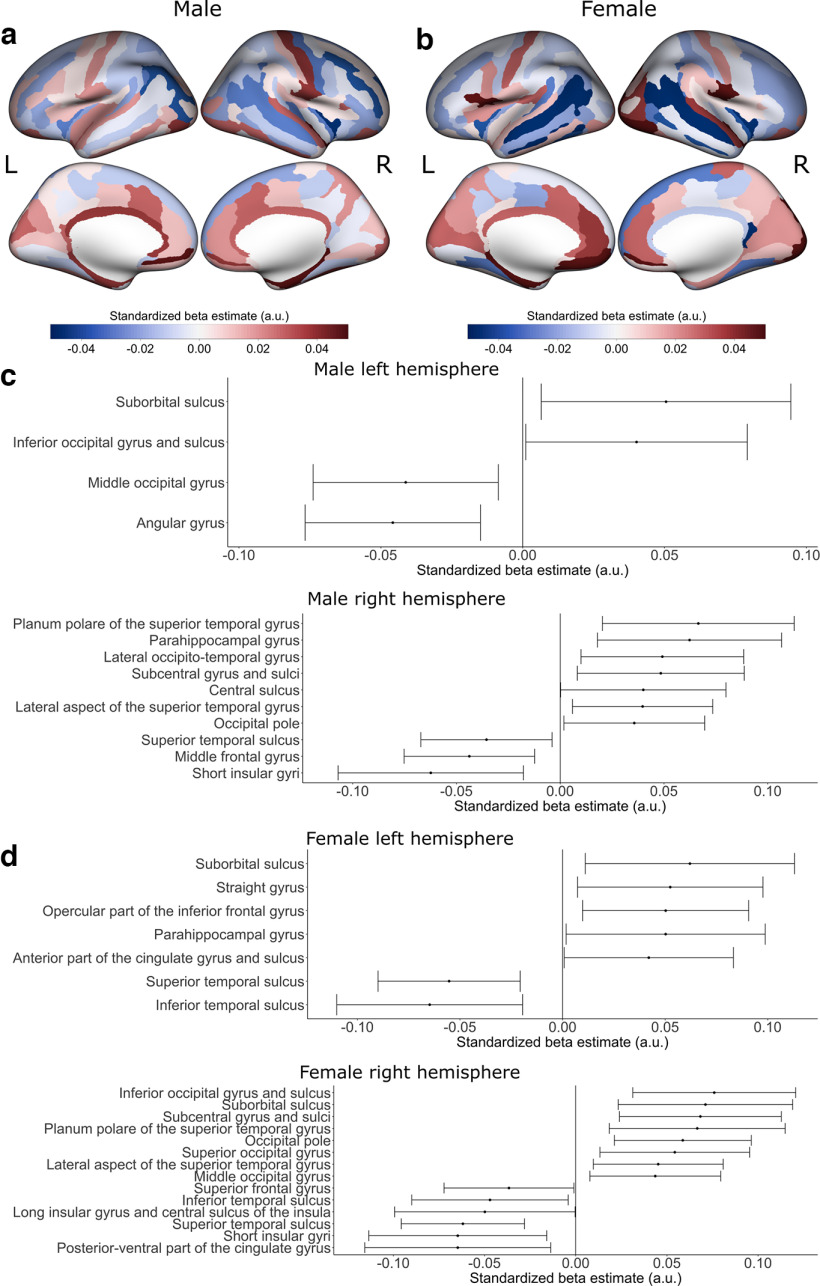
Preterm birth associations with cortical thickness stratified by sex. Positive β estimates are shown in red, indicating thicker cortical thickness with shorter gestational age, and negative β estimates are shown in blue, indicating thinner cortical thickness with shorter gestational age, for (***a***) males and (***b***) females. Estimated standardized βs of cortical regions whose 99% confidence interval do not overlap 0 are displayed for (top) left hemisphere cortical thickness and (bottom) right hemisphere cortical thickness for (***c***) males and (***d***) females. For the estimated standardized βs with their 99% confidence intervals for all cortical regions, refer to Extended Data Figure 7-1. a.u. = arbitrary units. L = left hemisphere; R = right hemisphere.

**Figure 8.**
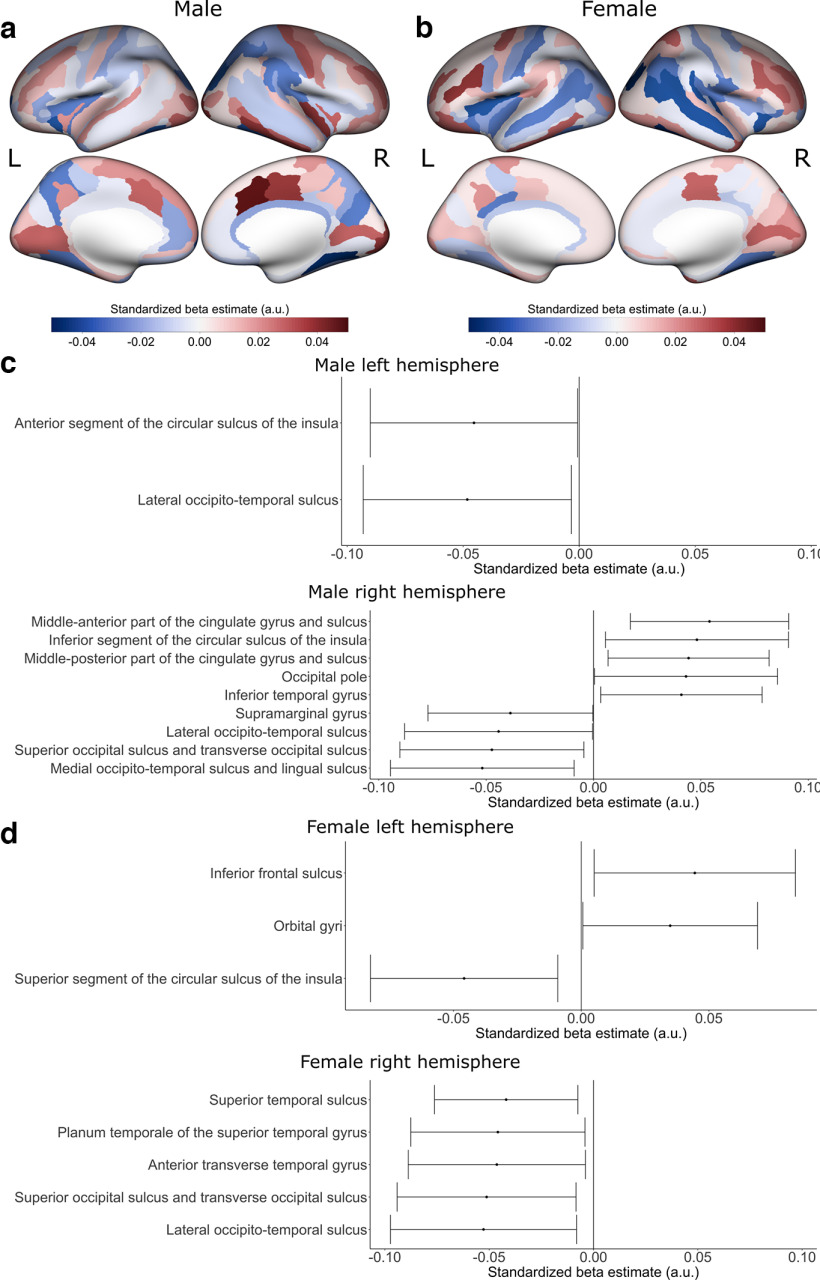
Preterm birth associations with cortical surface area stratified by sex. Positive β estimates are shown in red, indicating greater surface area with shorter gestational age, and negative β estimates are shown in blue, indicating smaller surface with shorter gestational age, for (***a***) males and (***b***) females. Estimated standardized βs of cortical regions whose 99% confidence interval do not overlap 0 are displayed for (top) left hemisphere surface area and (bottom) right hemisphere surface area for (***c***) males and (***d***) females. For the estimated standardized βs with their 99% confidence intervals for all cortical regions, refer to Extended Data Figure 8-1. a.u. = arbitrary units. L = left hemisphere; R = right hemisphere.

The size of the ABCD sample allowed for sex-stratified and sex-interaction analyses that are less feasible in smaller cohorts. Sex-stratified CT and SA models showed similar effects of PTB in boys and girls (Figs. 7, 8; Extended Data Figs. 7-1, 8-1). A very small number of regions had high confidence for an interaction between sex and PTB for CT (right middle occipital gyrus: b = −0.05 [−0.09, −0.003]), or SA (right short insular gyri: b = 0.05 [0.003, 0.11]). No high confidence interactions between sex and PTB were found for subcortical regions.

10.1523/ENEURO.0196-22.2023.f7-1 Extended Data Figure 7-1 Preterm birth associations with cortical thickness in (***a***) males and (***b***) females in all cortical regions. Estimated standardized βs are displayed with their 99% confidence intervals separately for the (top) left hemisphere cortical thickness and the (bottom) right hemisphere cortical thickness. a.u. = arbitrary units. Download Figure 7-1, TIF file.

10.1523/ENEURO.0196-22.2023.f8-1 Extended Data Figure 8-1 Preterm birth associations with cortical surface area in (***a***) males and (***b***) females in all cortical regions. Estimated standardized βs are displayed with their 99% confidence intervals separately for the (top) left hemisphere surface area and the (bottom) right hemisphere surface area. a.u. = arbitrary units. Download Figure 8-1, TIF file.

### Generalizability for findings from the discovery to the replication sample

**Figure 9.**
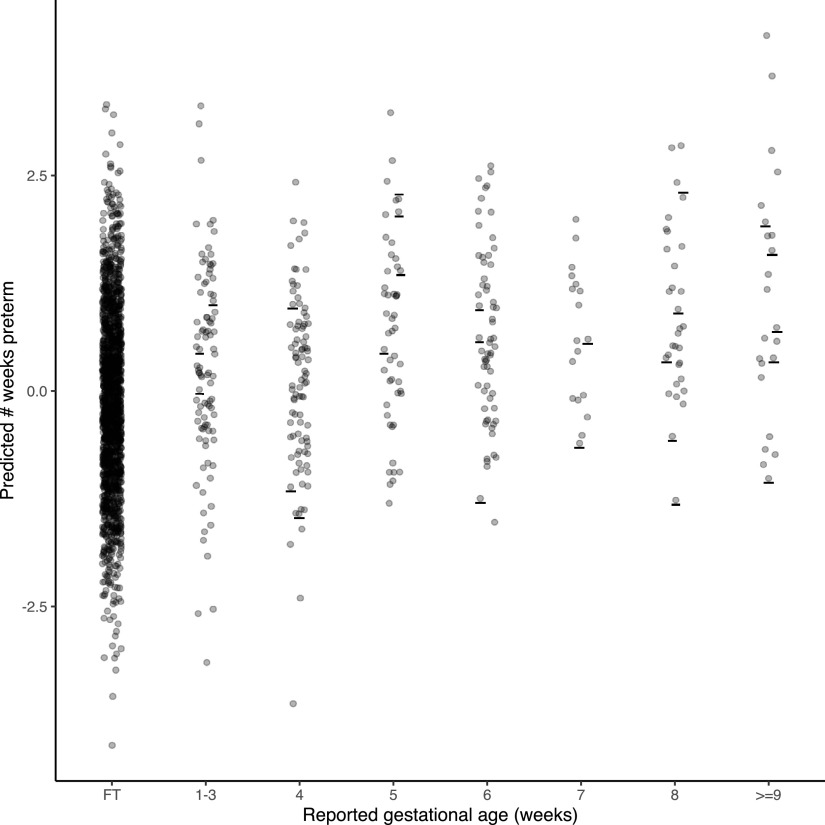
Prediction of preterm weeks in the holdout replication sample using cortical thickness parameter estimates from the discovery sample. We note that prediction is relative and centered around zero because only effects of regional cortical thickness were included. Predicted relative preterm weeks correlates with reported preterm weeks at *r*_s_ = 0.20 [0.16, 0.24].

We used high-confidence parameter estimates from models that linearly controlled for brain-size effects in the discovery sample to predict weeks born preterm in the replication sample and, using Spearman correlations (r_s_), compared predicted weeks born preterm to actual weeks born preterm. We found moderate prediction using cortical thickness, with predicted weeks born preterm correlated with actual preterm weeks at *r*_s_ = 0.20 [0.16, 0.24] (predicted vs actual values in Fig. 9). Surface area had a substantially lower association *r*_s_ = 0.05 [0.008, 0.09]. Relative to CT, associations were lower for subcortical (*r*_s_ = 0.09 [0.05, 0.14]), cerebellar (*r*_s_ = 0.11 [0.07, 0.15]), and ventricular regions (*r*_s_ = 0.02 [−0.02, 0.06]). Including all parameters in one predictive model showed the same association as that of CT alone (*r*_s_ = 0.20 [0.16, 0.24]).

## Discussion

This study leveraged the large and diverse ABCD sample to comprehensively characterize long-term effects of PTB on brain structure in late childhood using an estimation statistics framework. Our main findings were that PTB is associated with a pattern of relative cortical thinning around dorsal prefrontal and temporoparietal junction regions and thickening of medial prefrontal and occipital regions when controlling for mean hemispheric CT. Surface area was reduced in ventral visual regions and increased in dorsal prefrontal, mid-cingulate, and anterior ventral-temporal regions. We found that absolute volumes were smaller in subcortical regions in PTB, but the effects of PTB were attenuated when total brain volume was taken into account. In cortical regions, effects appeared to be partly mediated by birthweight. We found largely overlapping effects in boys and girls and limited evidence for interaction effects between sex and weeks born preterm. Finally, we show that effects estimated in the discovery sample predicted gestational age in the replication sample with a small-to-moderate effect size (*r*_s_ = 0.20), and that prediction was strongest for CT relative to other structural parameters.

Importantly, results are presented as effect sizes with confidence intervals, which helps to clarify both that effects for individual regions are relatively small and that regions where we found high-confidence of non-zero effects have confidence intervals that overlap substantially with lower-confidence neighbors, emphasizing the importance of considering a pattern of effects rather than hard boundaries defined by *p*-values.

Although effect sizes are small, both absolute and relative CT findings (models uncontrolled and controlled for mean hemispheric CT, respectively) concur with several previous studies that have reported thinning in temporal and occipitoparietal regions in children born preterm (Martinussen et al., 2005; Nagy et al., 2011; Skranes et al., 2012; Bjuland et al., 2013; Sølsnes et al., 2015; Sripada et al., 2018; Pascoe et al., 2019; Schmitz‐Koep et al., 2020). We further found thinning of dorsal prefrontal regions. As there is vulnerability to injury in peri-ventricular white matter related to PTB, these effects may be secondary to this injury as they occur in cortical regions linked by anterior-posterior white matter tracts such as the superior longitudinal fasciculus (Volpe, 2009). Models controlling for mean CT further showed thickening of some occipital and orbitofrontal regions, findings which have also been reported previously (Kelly et al., 2014; Sølsnes et al., 2015; Sripada et al., 2018).

In typically developing children, cortical SA undergoes a distinct developmental trajectory (Krongold et al., 2017) and has different regional heritability (Panizzon et al., 2009) than CT, motivating separate consideration of these cortical parameters. We found fewer high confidence effects for SA, relative to CT. We also noted that the effects on SA were more prominent in uncontrolled models and strongly attenuated in models controlling for total hemispheric SA. Further, SA was not a robust predictor of GA in the replication sample. These findings are perhaps not surprising given the inconsistent findings related to SA in the prior literature (Skranes et al., 2013; Grunewaldt et al., 2014; Sølsnes et al., 2015; Zhang et al., 2015; Mürner-Lavanchy et al., 2018; Hasler et al., 2020; Sripada et al., 2018; Young et al., 2020). Considering the pattern of effects for SA and CT, we see that in both there are reductions in children with PTB in temporoparietal regions, but estimates diverge in several other regions. CT estimates point to reductions in dorsal prefrontal regions, whereas SA estimates suggest increases in dorsolateral prefrontal regions. SA showed a reduction in bilateral ventral visual regions, which were not seen for CT. This effect has been previously reported in a small number of studies (Sripada et al., 2018; Hasler et al., 2020).

Somewhat surprisingly, we did not find strong evidence for enlarged ventricles, though we note that effect sizes were positive, as would be expected from previous literature (Stewart et al., 1999; Cooke and Abernethy, 1999; Nosarti et al., 2002; Kesler et al., 2004). Ventricle enlargement is likely secondary to perinatal injury such as intraventricular hemorrhage (Brouwer et al., 2016), and thus may only be present in a subset of children. The lack of findings here may be an indication that the PTB children in the more sociodemographically representative ABCD sample are relatively less affected than very PTB or extremely low birthweight children recruited into research studies through perinatal follow-up programs; indeed, a very small number of children in the present study were born at the gestational ages with the highest risk. Perhaps similarly, we found few subcortical regions with strongly reduced volumes in children born preterm relative to FTB, although we note that effect size estimates for subcortical volumes were more negative in analyses that did not control for total brain volume and became less negative when controlled for total brain volume. This suggests that, in addition to sample characteristics, variation in findings across studies may partially relate to whether total brain volume was included as a control variable. Cerebellar white matter volume was more prominently reduced than cerebellar gray matter volume in the liberal QC sample, though we note that white matter effects were attenuated in the stringent QC sample, suggesting caution in interpreting this effect.

Birth complications secondary to infection and inflammation are often seen in preterm born neonates which may confound the effects of preterm birth on brain structure in children (Reiss et al., 2022). To consider this potential confounding factor, we conducted a set of analyses that controlled for birth complications that required the participants to be hospitalized for a month after being born. Differences in associations with GA relative to analyses that did not include this covariate were primarily noted for surface area. This may indicate a reduced specificity of associations with surface area, which perhaps contributes to the limited predictive ability seen in the holdout sample. We note that birth complications as recorded here did not specify the type of insult which may contribute to limited associations with brain structure and makes findings related to this variable challenging to interpret. In future work it will be important to parse perinatal complications further to gain more insight into long-term effects on brain structure.

A relatively unique contribution of this work is that the PTB group was heavily weighted to moderate/late preterm birth. A growing number of studies suggest that although very/extremely preterm and low birthweight infants are at highest risk for adverse outcomes, for some outcomes there is a graded risk based on gestational age, and risk may be exacerbated by environmental factors (Romeo et al., 2010, 2016; Potijk et al., 2013; Brumbaugh et al., 2016; Stene-Larsen et al., 2016). We find here that the pattern of effects on CT was similar for moderate and very PTB groups, though with smaller effect sizes for children born moderately preterm. As moderate PTB affects proportionally more children than very PTB, this is an important population from a public health perspective (Natarajan and Shankaran, 2016). Our findings of graded brain structure alterations support calls for more research and targeted follow-up of the moderate/late PTB population to support optimal childhood outcome (Cheong et al., 2017; Favrais and Saliba, 2019).

A previous analysis of ABCD data that covaried brain structure with cognitive, clinical, behavioral, and sociodemographic variables found that the strongest mode of variation related perinatal factors and obstetric complications to brain morphology, including regions that parallel findings here in occipital, orbitofrontal, temporal and parietal regions (Alnæs et al., 2020). A second ABCD study using related methodology (Modabbernia et al., 2021) also identified a mode of brain-phenotype variation that was related to birthweight, prematurity, and twin birth. Together this work shows the relative importance of perinatal factors on long-term brain structure and population-level variation, relative to the many other factors included in these analyses and underscores the importance of studies with large sample sizes such as ABCD to enable realistic estimates of population variation.

Preterm birth affects the brain in several ways. Developmental timing is altered, such that late developing brain structures undergo maturation under markedly different conditions ex-utero. There is increased risk of perinatal injury such as periventricular leukomalacia (Volpe, 2009). Infants born preterm are exposed to a number of perinatal medical procedures, which have in turn been linked with brain changes such as smaller subcortical volumes (Chau et al., 2019). Further, infants born preterm are smaller than full-term born infants and birthweight shows robust long-term associations with brain structure (Walhovd et al., 2012) as well as cognition (Matte et al., 2001; Newcombe et al., 2007) and behavior (Pettersson et al., 2015; Lim et al., 2018). We found in our analysis that many high-confidence effects could be partly mediated by birthweight. Effects of PTB on the brain are known to be heterogenous and this finding further underscores that alterations linked with PTB may have varied etiology. The ABCD sample likely does not capture many children with severe perinatal brain injury secondary to PTB. Therefore, it is perhaps not surprising that effects are modest, and birthweight plays an important role in mediating effects in this sample.

Several studies have shown that boys are at elevated higher risk for adverse outcomes following PTB (Whitfield et al., 1997; Hindmarsh et al., 2000; Wood et al., 2005; Hintz et al., 2006; Young et al., 2016; Urben et al., 2017). In moderate or late preterm birth, a small number of studies have instead suggested that girls are at a higher risk for behavioral challenges (Stene-Larsen et al., 2016; Ask et al., 2018). Here, we found largely similar effects of PTB on the brain structure of girls and boys in sex-stratified analyses and that there were limited interactions between sex and PTB on brain structure. Male vulnerability may be more evident in samples of children who were born very PT; the ABCD sample included a higher proportion of moderate PTB children than most studies that have examined brain structure in PTB children.

A goal of this analysis was to use a large sample to help address some inconsistencies in the literature on PTB. Indeed, recent work has reinforced that in brain-wide association studies, small samples can lead to inflated estimates of effect sizes, and that for associations between brain and behavioral outcomes, sample sizes in the thousands may be required (Marek et al., 2022). Work in the ABCD sample has further suggested that expectations about effect sizes for associations between psychological variables may need to be recalibrated as median effect sizes are much smaller when examined in this large sample (Owens et al., 2021). To this end, we note that by focusing on effect size estimates and confidence intervals, results presented here help to mitigate the challenges that regions falling just above or below statistical thresholds in different studies can give the misleading impression of divergent findings. Further, by conducting analyses with and without adjusting for brain size, we observed that the pattern of effects was similar whether or not brain size adjustment was done, but brain size adjustment tended to positively shift effect estimates of preterm birth, such that positive effects were relatively amplified, and negative effects were smaller. In general, although adjusting is intended to help to give more specific regional effects, it is not clear whether absolute or relative volumes are more important for understanding behavioral outcomes. Previous PTB literature has suggested more convergent effects in CT relative to SA findings, which is supported by findings here of weaker SA effects that did not generalize to a holdout sample. Finally, reports on interactions between sex and PTB have been inconsistent, our results suggest that in the population considered here, brain structural differences are not strongly affected by sex. However, this is noted with the caveat that our sample is largely moderately rather than very PTB children, which may limit generalization to more affected populations.

PTB is known to impact cognitive challenges including general intellectual functioning (Twilhaar et al., 2018), attention (Bogičević et al., 2021), language and reading (Taskila et al., 2022), as well as children’s behavior challenges (Burnett et al., 2019) and longer-term mental health risks (Vanes et al., 2022). Although directly relating brain structure to behavior is beyond the scope of the present study, future work can consider whether and to what extent brain structural alterations mediate cognitive outcomes. Indeed, several smaller cohort studies have suggested a mediating influence of both gray (Hedderich et al., 2019, 2020; Schmitz‐Koep et al., 2020) and white (Nosarti et al., 2008; Berndt et al., 2019) matter structure on cognitive outcomes following very PTB.

While the strengths of this study include a large and sociodemographically diverse sample, a narrow age range limiting the influence of age effects, and multiparameter whole-brain examination, there are several notable limitations. The first is that the ABCD study did not set out to recruit children born preterm; therefore, the sample size of PTB children is relatively small, especially for lower GA groups whose brains are more affected. Further, perinatal variables were collected through self-report rather than chart review and many potential influences on perinatal brain development were not considered here (e.g., hospitalization, medical procedures). We note that several studies have found that painful procedures around the time of birth associates with brain structure and outcomes following preterm birth (Brummelte et al., 2012; Duerden et al., 2018; Tortora et al., 2019), and we note that the absence of detailed clinical data are therefore a limitation here.

To consider the impact of harmonization method, we include results from an analysis using ComBat (Fortin et al., 2017) to examine association between PTB and cortical thickness (Extended Data Fig. 2-6). We note no substantive changes to the primary analysis in which we chose to address site-related confounds as a random effect in the mixed-effects model (Fig. 2). We ultimately decided to proceed with including site as a random effect in our models because (1) it was the suggested method within the Data Exploration and Analysis Portal provided by the ABCD study developers (ABCD Study) and (2) it enabled us to use a nested approach to deal with family ID as well to account for any clustering effects that may have come from sibling and twin data.

We used ROI-by-ROI values rather than a vertex-wise modeling, potentially leading to attenuation of effects that do not span entire regions. Although this is a childhood sample and therefore the brain is developing, analyses were conducted on cross-sectional rather than longitudinal data. While much of the work to date has been cross-sectional, a small number of longitudinal studies across childhood and adolescence have suggested relatively parallel development of structure across late childhood or adolescence (Sripada et al., 2018; Thompson et al., 2020; Vandewouw et al., 2020), i.e., few and/or small differences in developmental trajectories. A series of studies scanning the same individuals at different time points showed strikingly similar group differences across timepoints suggesting relative stability of structural differences (Sølsnes et al., 2015; Rimol et al., 2016; Dewey et al., 2019). For this reason, we anticipate that trajectory differences may be small and that large samples like ABCD may be useful for resolving differences.

In sum, this study replicates previous findings of a pattern of fronto-temporoparietal thinning in PTB and thickening of occipital and medial prefrontal regions in a large and sociodemographically diverse late childhood sample. We extended this work by showing region-wise effect sizes with confidence intervals, finding that in this large population sample these effects may be partly mediated by birthweight and do not differ substantially between sexes. CT in high-confidence regions predicted GA in a “replication” sample, demonstrating generalizability of findings and the relative consistency of differences in cortical thickness in PTB children across samples.

10.1523/ENEURO.0196-22.2023.ed1 Extended Data 1 All code used for the statistical analyses described is freely available at https://github.com/BrayNeuroimagingLab/BNL_open/tree/main/abcdPTB. Download Extended Data 1, ZIP file.

## Synthesis

Reviewing Editor: Nicholas J. Priebe, University of Texas at Austin Decisions are customarily a result of the Reviewing Editor and the peer reviewers coming together and discussing their recommendations until a consensus is reached. When revisions are invited, a fact-based synthesis statement explaining their decision and outlining what is needed to prepare a revision will be listed below. The following reviewer(s) agreed to reveal their identity: NONE.

Dear authors,

Thanks for your work on the neuroanatomical correlates of preterm birth. The article is clear and well-written, the analyses are robust (save for a few issues detailed below) and, most importantly, the findings are important to address inconsistencies in the literature. I enclose the reviewers’ comments below, which must be addressed before the article can be considered for publication.

Major:

- There are discrepancies in cortical thickness and surface area findings mentioned in the Introduction. Could the authors elaborate in the Discussion how their findings address these discrepancies in the literature?

- It has been reported that those born preterm have differences in development and cognition compared to those born fullterm. Could the authors test how the anatomical differences described here may relate to the cognitive changes described in the literature around PTB? If the data are available (and they should be in such a comprehensive dataset), linking differences in PTB-related brain anatomy and psychological functioning would greatly strengthen the paper and leave less room for speculation regarding the practical implications of the results.

- Throughout the manuscript, the authors distinguish “mild” and “very” PTB and either spell it out or use the abbreviations MPTB and VPTB respectively. It seems these abbreviations are used sparingly and I would recommend spelling out “mild” and “very” as the abbreviations got a bit confusing.

- In preterm births, particularly those < 36 weeks GA, it is likely these newborns were in the newborn intensive care unit and/or under anesthesia. Is there a chance that this early-life anesthesia exposure influenced any of the dependent variables? If the data are available, the authors should control for it statistically, or at the very least qualitatively comment on data from anesthetized participants if the number is too low. If these data are not available, the authors could briefly address this in the Discussion.

- In Figure 5, it seems that there are multiple regions completely mediated by birthweight (in yellow) for both CT and SA.

- For sex differences in CT (Figure 6), it appears there are differences in males and females particularly in the positive effects seen in temporal cortex. Effects in OFC seem similar. Could the authors address this particular difference?

- How might these changes in cortical thickness, SA, etc relate to fiber changes in PTB?

- Can the authors account for the discrepancy between regions that show greater SA in PTB yet thinner cortical thickness (like in dlPFC)? What effects may that have on cognition or connectivity patterns?

- It is important to point out in the introduction why controlling for total brain volume is important when reporting regional differences - if not controlled for, it’s impossible to tell whether results are just a by-product of smaller head size.

- A histogram of the GA would be helpful for the “Preterm birth analysis in the discovery sample” section.

- Once the linear mixed models were fit were the residuals normally distributed, as is required by linear mixed models? If this is not the case, general linear modeling with a specific distribution should be used depending on the degree of violation.

- On a related note, was the random effects structure of the models maximized, as recommended for reproducibility? See Barr et al. (2013)

- Have the authors considered using ComBat harmonization to control for site effects instead of using a covariate in the analysis? ComBat has been shown to be superior to the covariate method to address multisite data. Given that whole sites were assigned to the discovery and replication samples, sufficiently controlling for site effects is of utmost importance to this study.

- It would be helpful to also report any relations with the measures to control for total brain size with gestational age (perhaps in the supplement?).

- Figure 1 (and the subsequent figures) would be much easier to interpret if the regions whose confidence intervals crossed zero were masked out, instead of the reader having to figure out which regions shifted from blue to red. I appreciate reporting non-significant results and the authors focus on reporting effect sizes instead of p-values, and think it’s important to be transparent for all results, so perhaps these figures can either be (a) modified to include another column just showing the significant regions, with the non-significant regions masked out, or (b) be moved to the supplemental and only the significant regions highlighted in the main text.

- The recent paper by Marek et al (2022) discussing the sample size required for reproducible brain-wise associations, and how even samples in the thousands produce small effect sizes (as observed in this sufficiently powered study), would be a great addition to the discussion.

Minor comments:

- Please spell out abbreviations in figure captions

- It’s not clear what “relatively diverse” means in the abstract when describing the ABCD study.

- I think it would help to state whether the cortical thinning was present when controlling for total brain size in the abstract, since this is included for the subcortical effects.

- It would be helpful to repot the total number of brain regions in the atlas.

Reviewer references

- Barr, D. J., Levy, R., Scheepers, C., & Tily, H. J. (2013). Random effects structure for confirmatory hypothesis testing: Keep it maximal. Journal of Memory and Language, 68(3), 10.1016/j.jml.2012.11.001. https://doi.org/10.1016/j.jml.2012.11.001

- Marek, S., Tervo-Clemmens, B., Calabro, F. J., Montez, D. F., Kay, B. P., Hatoum, A. S., Donohue, M. R., Foran, W., Miller, R. L., Hendrickson, T. J., Malone, S. M., Kandala, S., Feczko, E., Miranda-Dominguez, O., Graham, A. M., Earl, E. A., Perrone, A. J., Cordova, M., Doyle, O., ... Dosenbach, N. U. F. (2022). Reproducible brain-wide association studies require thousands of individuals. Nature, 1-7. https://doi.org/10.1038/s41586-022-04492-9
