# Supplementary figures and images for: Long-Term Effects of Preterm Birth on Children’s Brain Structure: An Analysis of the Adolescent Brain Cognitive Development (ABCD) Study
Source: eNeuro. 2023 Jun 7;10(6):ENEURO.0196-22.2023. doi: 10.1523/ENEURO.0196-22.2023 (PMC10262676; doi:10.1523/ENEURO.0196-22.2023)

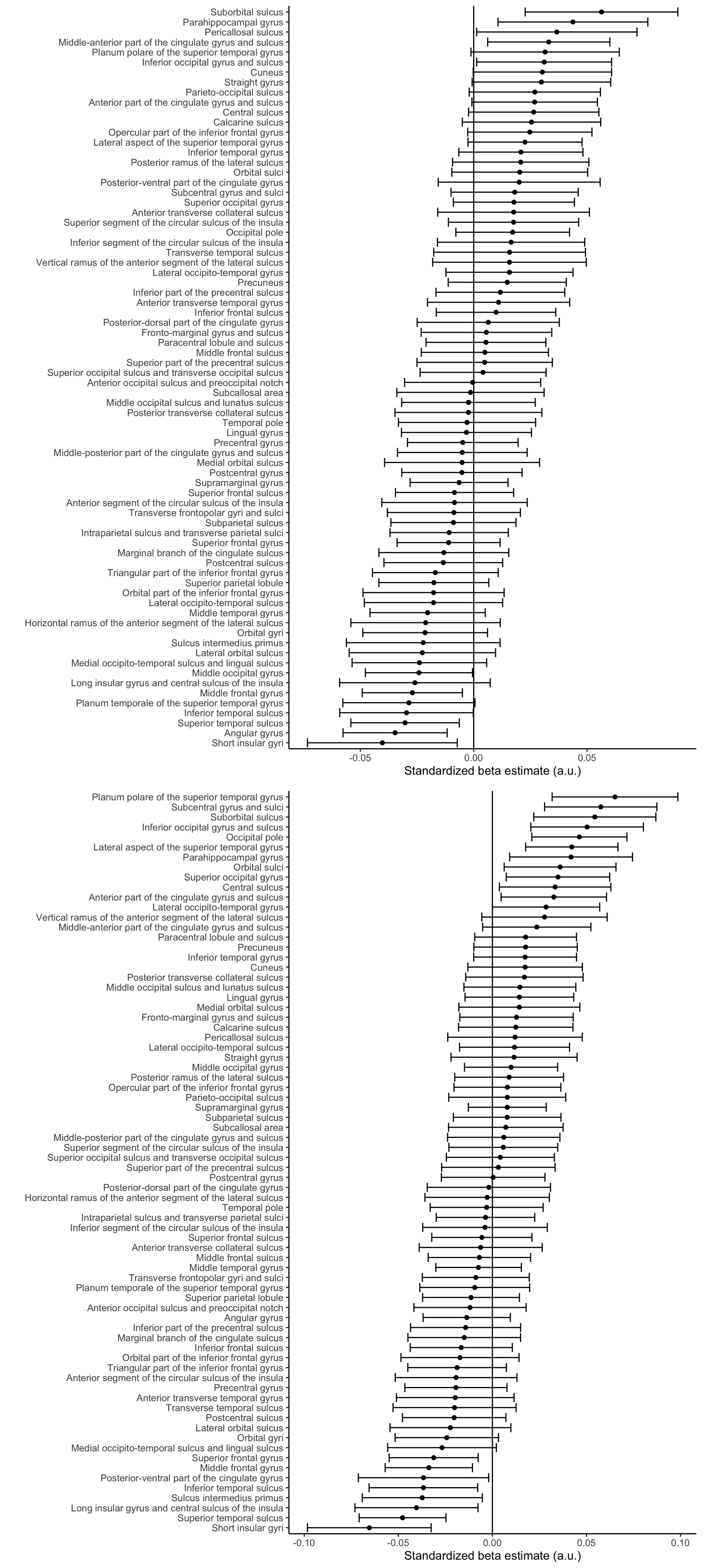

Supplement: Extended Data Figure 2-1 — Preterm birth associations with cortical thickness with a linear control for mean hemispheric cortical thickness in all cortical regions. Estimated standardized βs are displayed with their 99% confidence intervals separately for the (top) left hemisphere cortical thickness and the (bottom) right hemisphere cortical thickness. a.u. = arbitrary units. Download Figure 2-1, TIF file. [file enu-eN-NWR-0196-22-s04.tif]

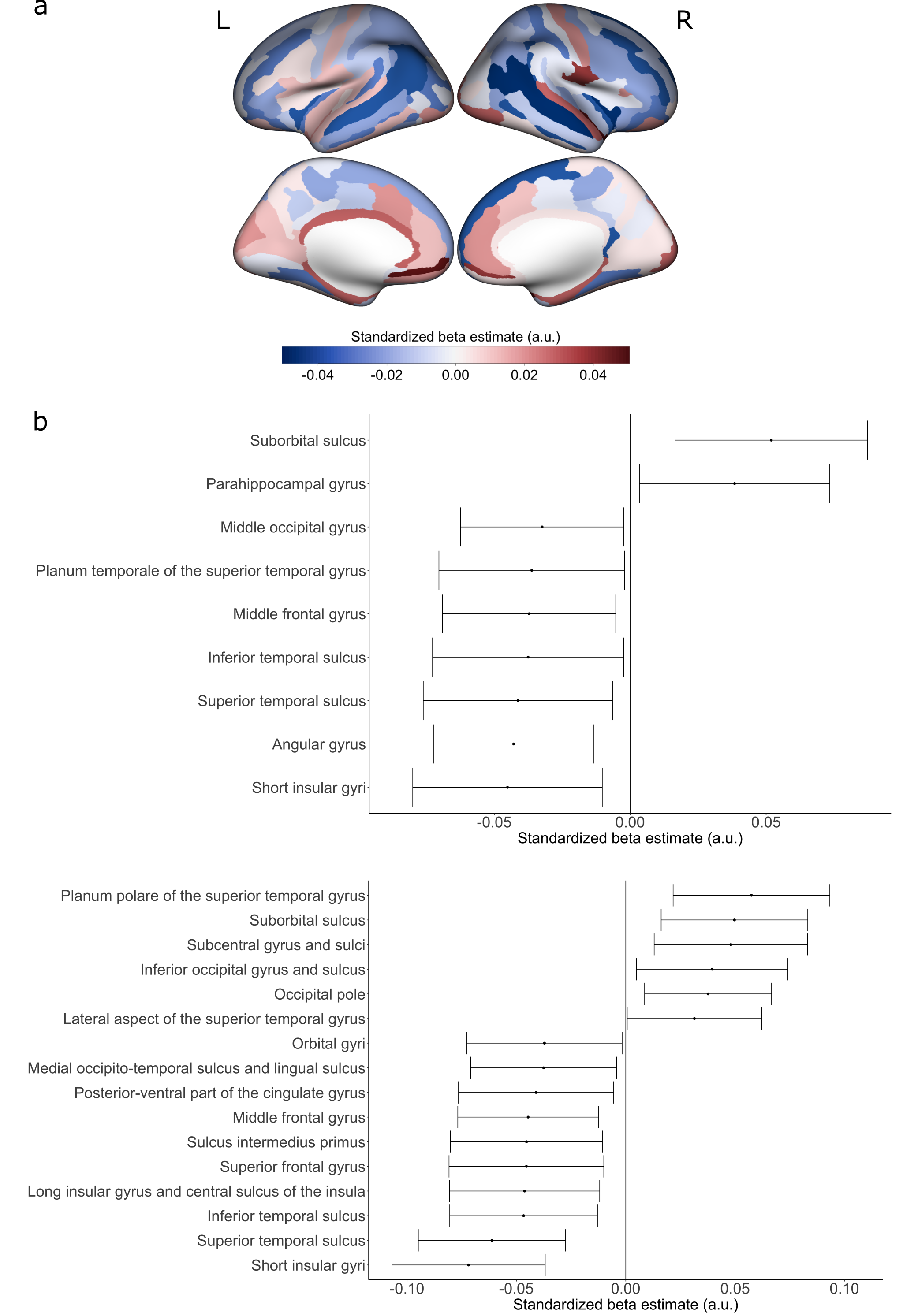

Supplement: Extended Data Figure 2-2 — Preterm birth associations with cortical thickness without a control for mean hemispheric cortical thickness. a, Positive β estimates are shown in red, indicating thicker cortical thickness with shorter gestational age. Negative β estimates are shown in blue, indicating thinner cortical thickness with shorter gestational age. b, Estimated standardized βs of cortical regions whose 99% confidence interval do not overlap 0 are displayed for (top) left hemisphere cortical thickness and (bottom) right hemisphere cortical thickness. a.u. = arbitrary units. Download Figure 2-2, TIF file. [file enu-eN-NWR-0196-22-s06.tif]

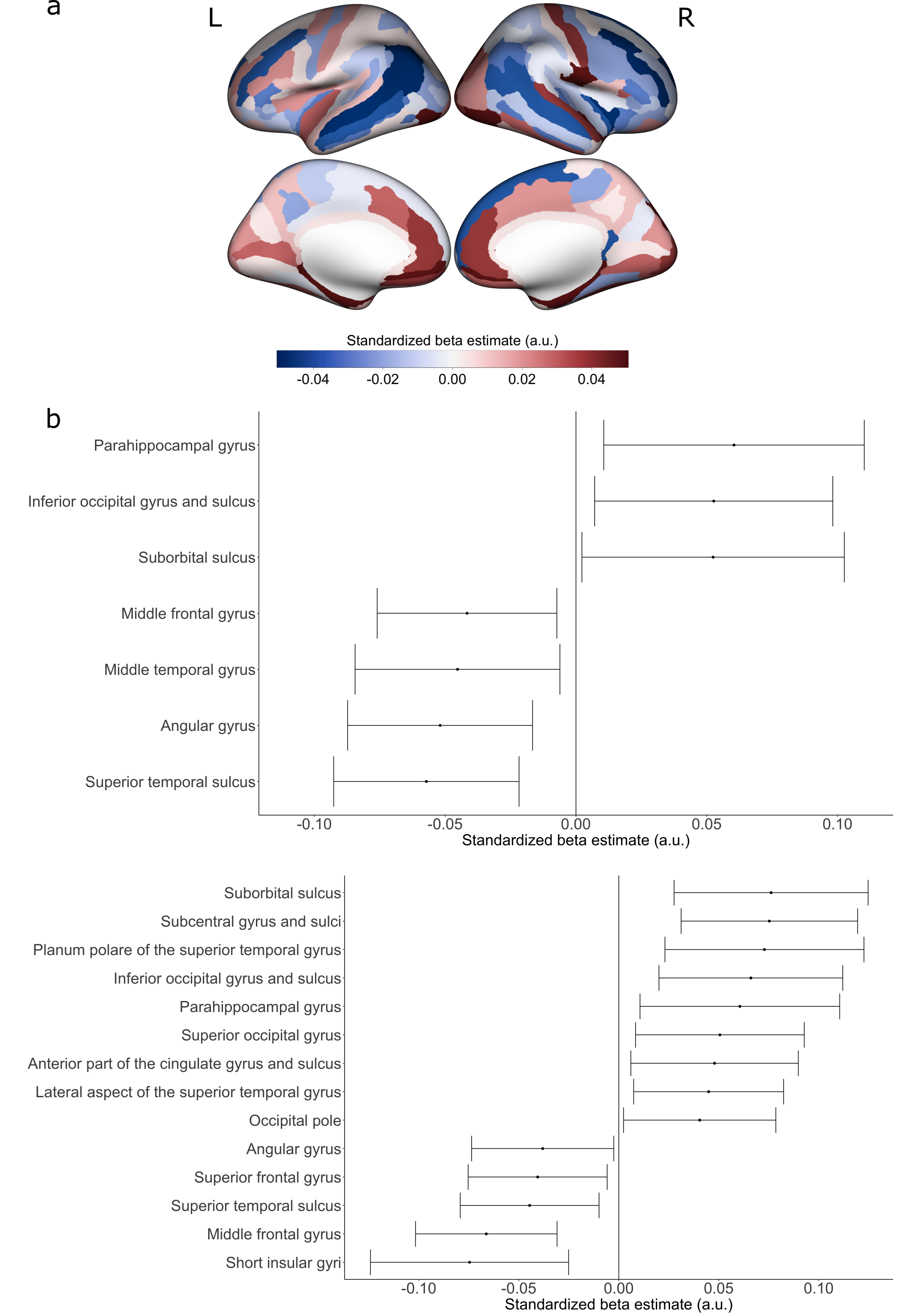

Supplement: Extended Data Figure 2-3 — Preterm birth associations with cortical thickness in stringent quality subsample. a, Positive β estimates are shown in red, indicating thicker cortical thickness with shorter gestational age. Negative β estimates are shown in blue, indicating thinner cortical thickness with shorter gestational age. b, Estimated standardized βs of cortical regions whose 99% confidence interval do not overlap 0 are displayed for (top) left hemisphere cortical thickness and (bottom) right hemisphere cortical thickness. a.u. = arbitrary units. Download Figure 2-3, TIF file. [file enu-eN-NWR-0196-22-s08.tif]

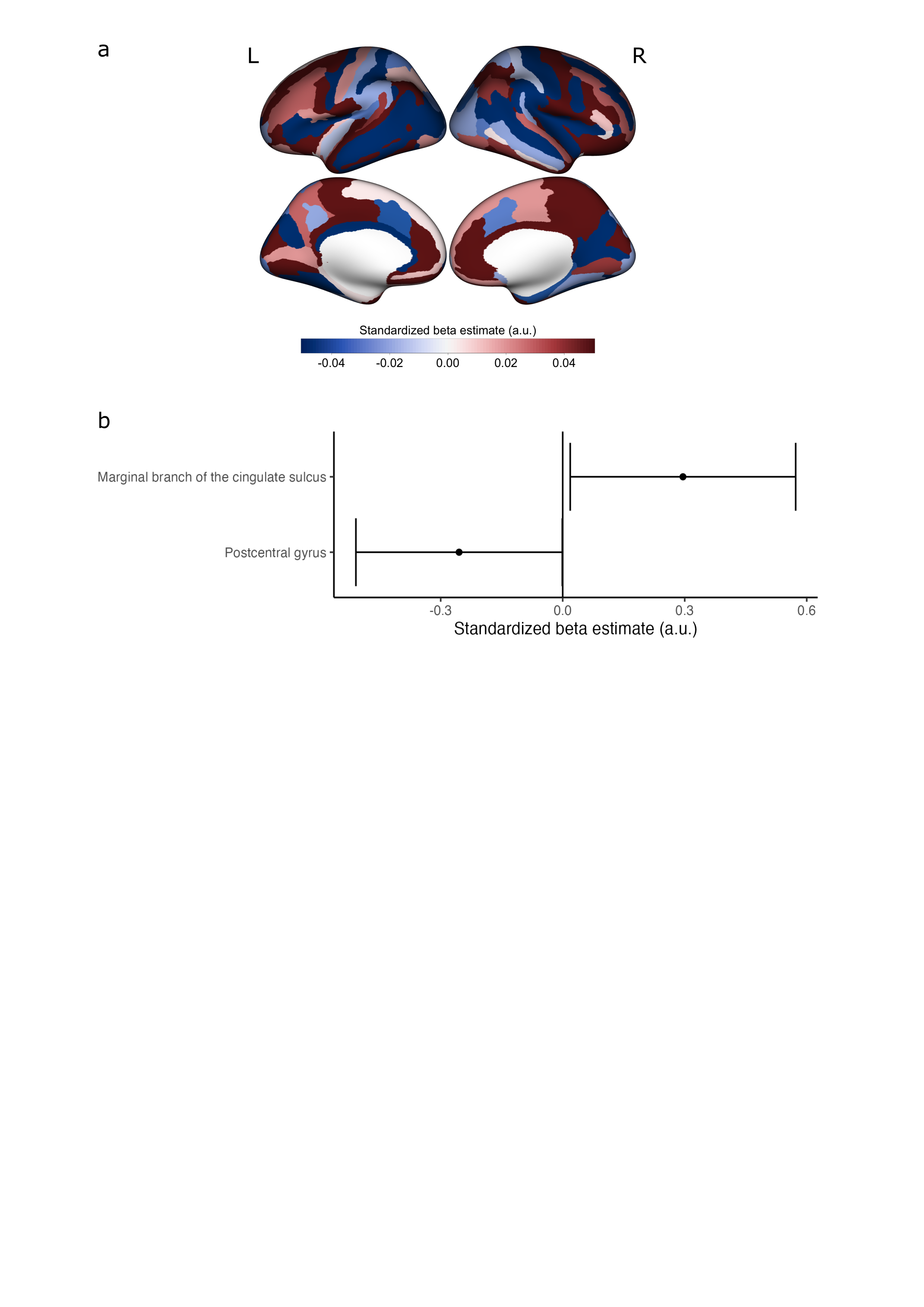

Supplement: Extended Data Figure 2-4 — Birth complication associations with cortical thickness. a, Positive β estimates are shown in red, indicating thicker cortical thickness in children who experienced a birth complication requiring hospital stay. Negative β estimates are shown in blue, indicating thinner cortical thickness in children who experienced a birth complication requiring hospital stay. b, Estimated standardized βs of cortical regions whose 99% confidence interval do not overlap 0 are displayed for right hemisphere cortical thickness; all estimated standardized βs of cortical regions in the left hemisphere had a 99% confidence interval that overlapped 0. a.u. = arbitrary units. Download Figure 2-4, TIF file. [file enu-eN-NWR-0196-22-s03.tif]

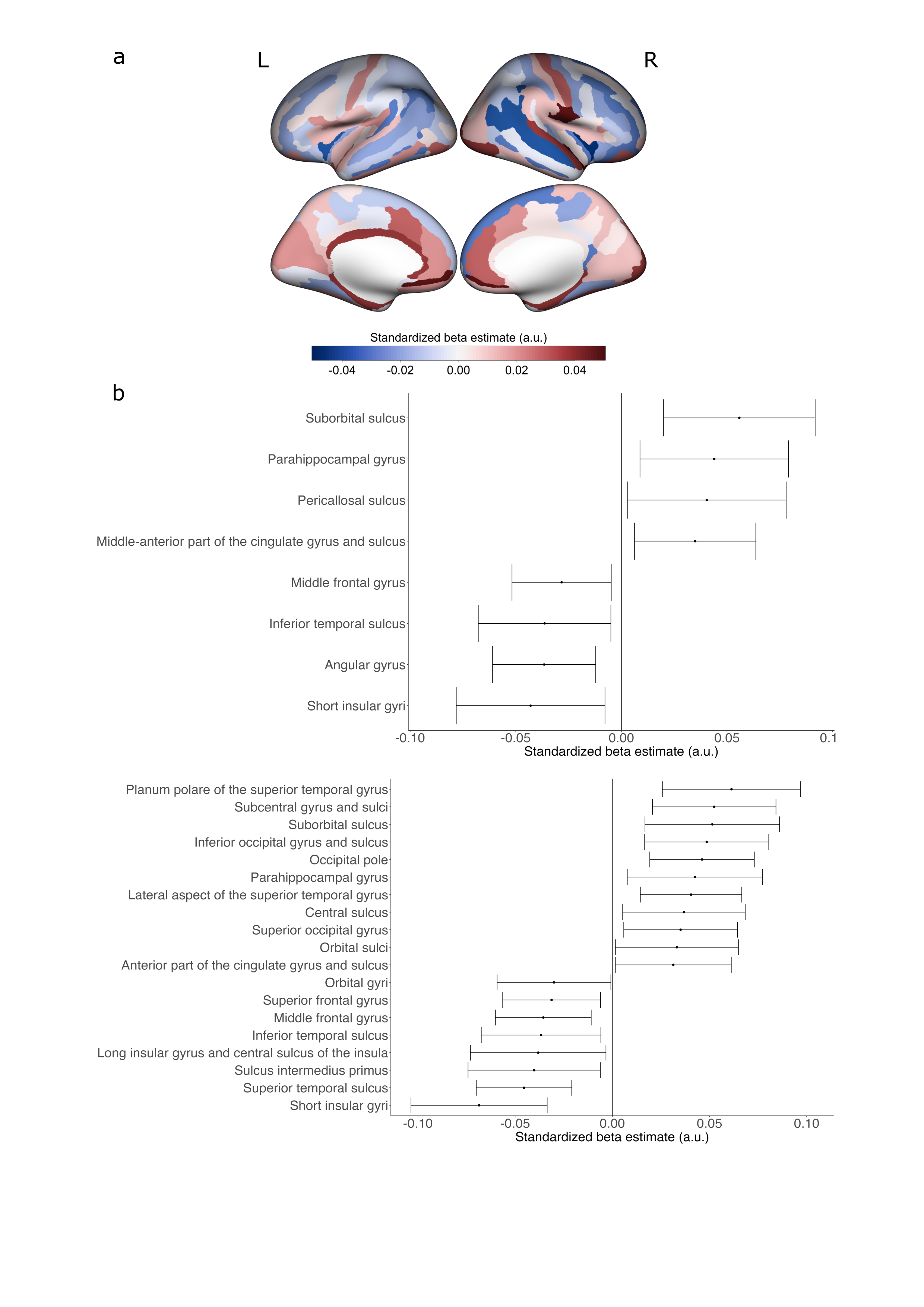

Supplement: Extended Data Figure 2-5 — Preterm birth associations with cortical thickness with a linear control for birth complications. a, Positive β estimates are shown in red, indicating thicker cortical thickness with shorter gestational age. Negative β estimates are shown in blue, indicating thinner cortical thickness with shorter gestational age. b, Estimated standardized βs of cortical regions whose 99% confidence interval do not overlap 0 are displayed for (top) left hemisphere cortical thickness and (bottom) right hemisphere cortical thickness. a.u. = arbitrary units. Download Figure 2-5, TIF file. [file enu-eN-NWR-0196-22-s05.tif]

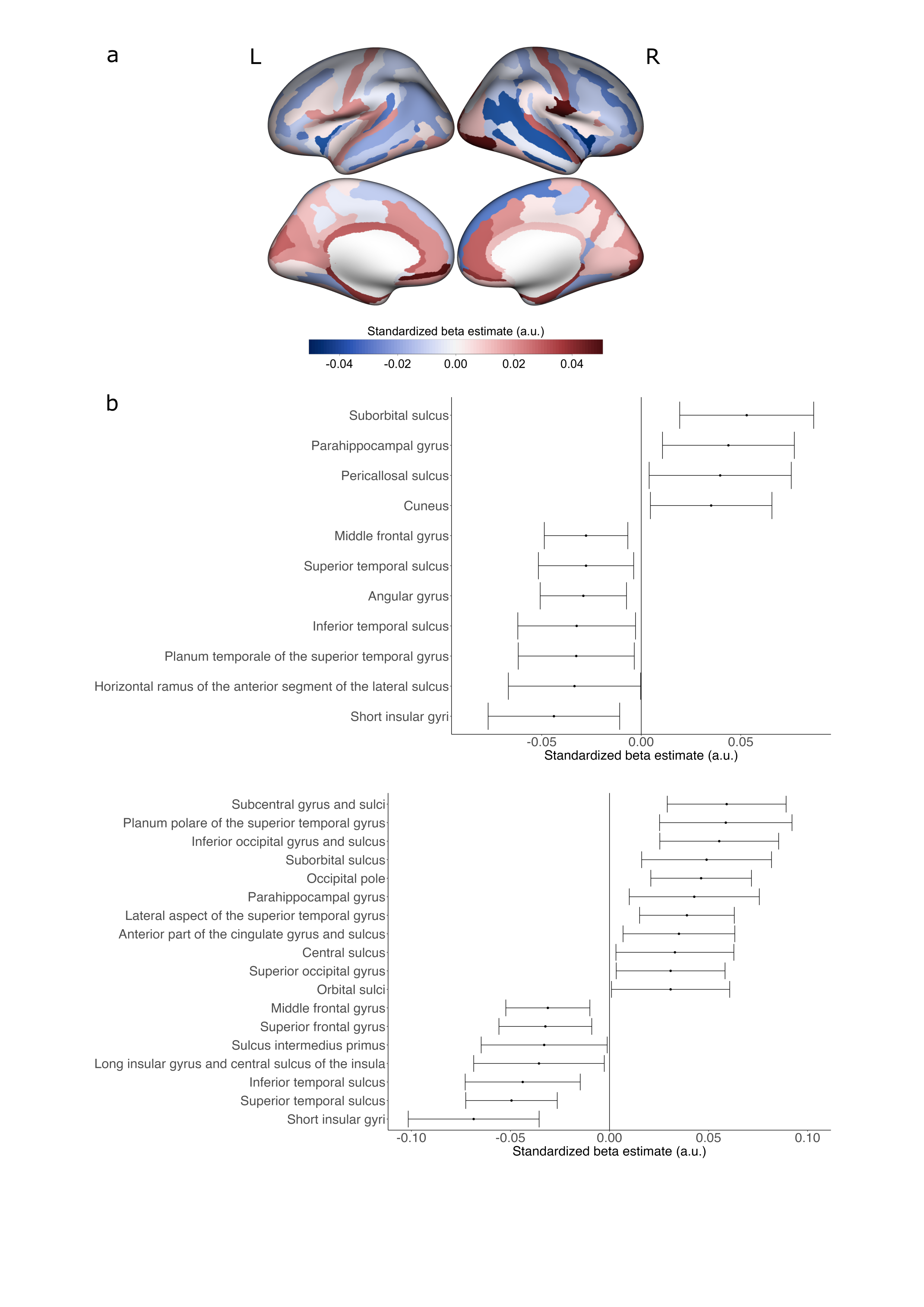

Supplement: Extended Data Figure 2-6 — Preterm birth associations with cortical thickness with a linear control for mean hemispheric cortical thickness using ComBat as the method of controlling variation between sites. a, Positive β estimates are shown in red, indicating thicker cortical thickness with shorter gestational age. Negative β estimates are shown in blue, indicating thinner cortical thickness with shorter gestational age. b, Estimated standardized βs of cortical regions whose 99% confidence interval do not overlap 0 are displayed for (top) left hemisphere cortical thickness and (bottom) right hemisphere cortical thickness. a.u. = arbitrary units. Download Figure 2-6, TIF file. [file enu-eN-NWR-0196-22-s07.tif]

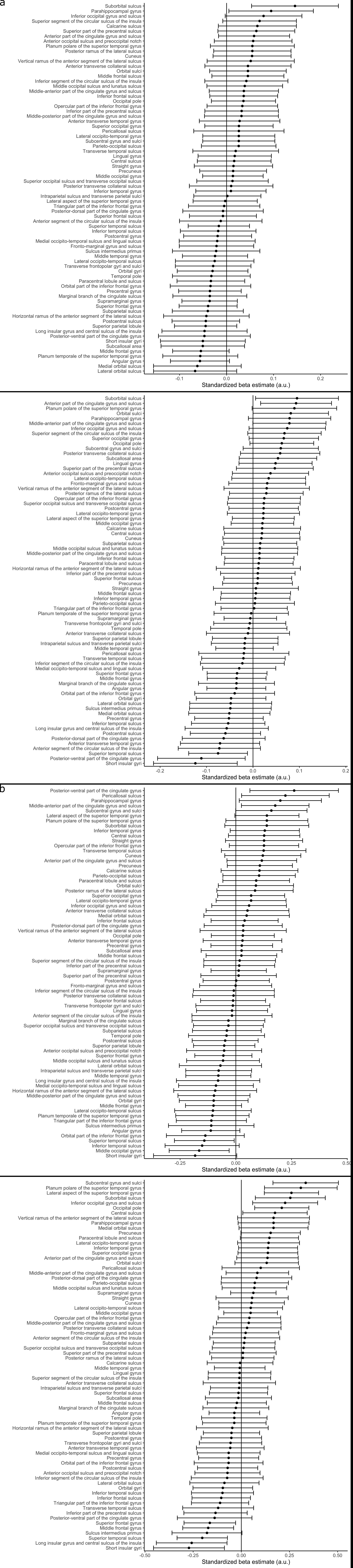

Supplement: Extended Data Figure 3-1 — Preterm birth associations with cortical thickness comparing (a) moderate preterm birth relative to full term birth and (b) very preterm birth relative to full term birth in all cortical regions. Estimated standardized βs are displayed with their 99% confidence intervals separately for the (top) left hemisphere cortical thickness and the (bottom) right hemisphere cortical thickness. a.u. = arbitrary units. Download Figure 3-1, TIF file. [file enu-eN-NWR-0196-22-s09.tif]

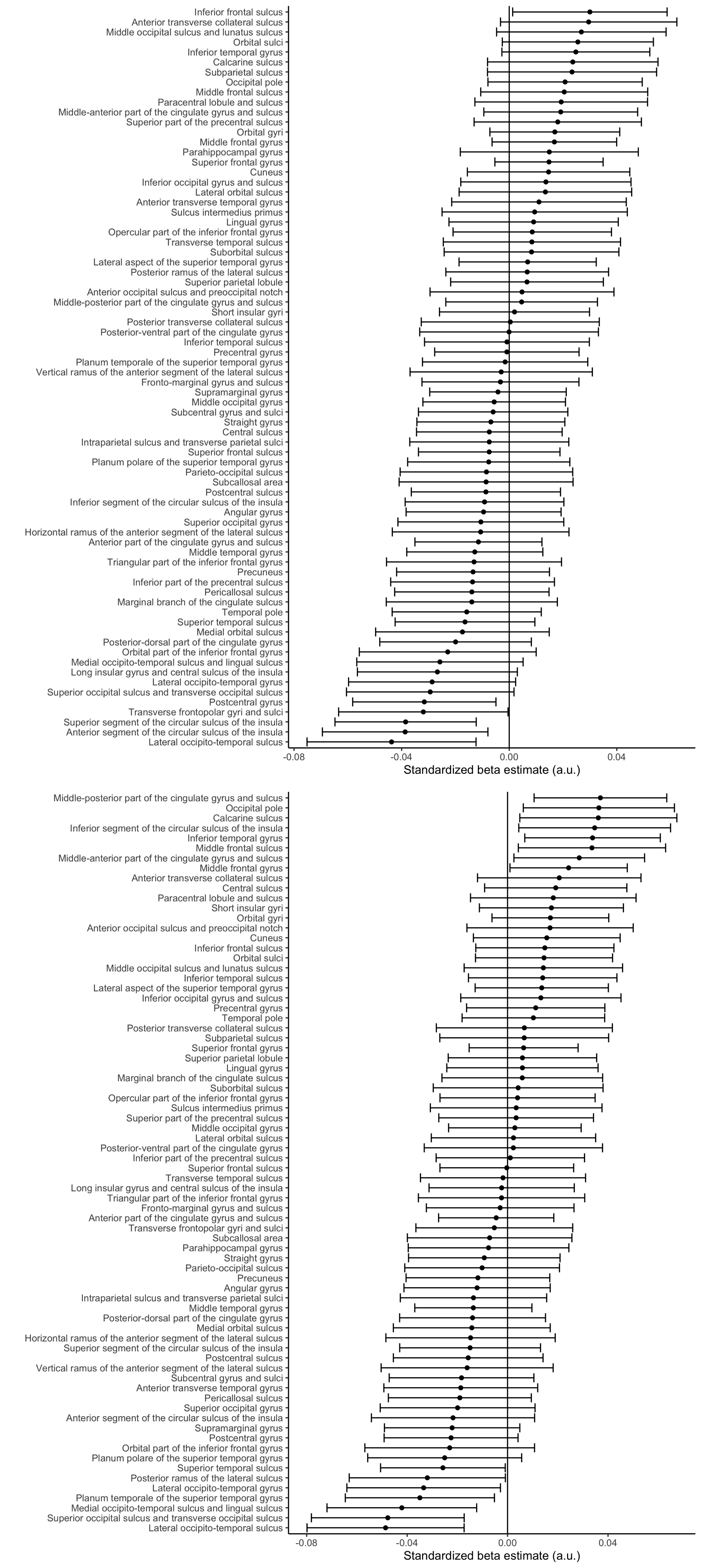

Supplement: Extended Data Figure 4-1 — Preterm birth associations with cortical surface area with a linear control for total hemispheric cortical surface area in all cortical regions. Estimated standardized βs are displayed with their 99% confidence intervals separately for the (top) left hemisphere surface area and the (bottom) right hemisphere surface area. a.u. = arbitrary units. Download Figure 4-1, TIF file. [file enu-eN-NWR-0196-22-s11.tif]

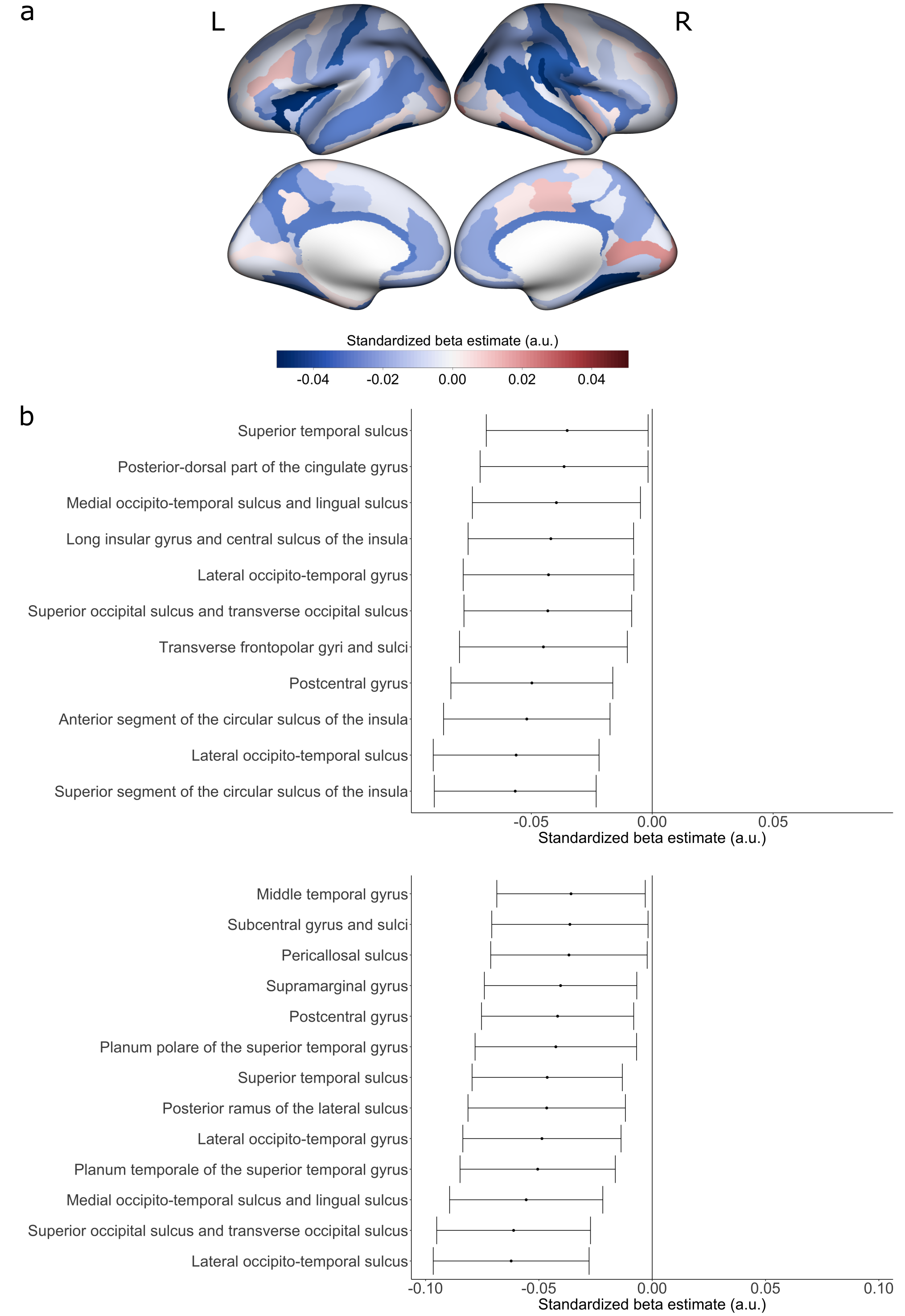

Supplement: Extended Data Figure 4-2 — Preterm birth associations with cortical surface area without a control for total hemispheric cortical surface area. a, Positive β estimates are shown in red, indicating greater surface area with shorter gestational age. Negative β estimates are shown in blue, indicating smaller surface area with shorter gestational age. b, Estimated standardized βs of cortical regions whose 99% confidence interval do not overlap 0 are displayed for (top) left hemisphere surface area and (bottom) right hemisphere surface area. a.u. = arbitrary units. Download Figure 4-2, TIF file. [file enu-eN-NWR-0196-22-s13.tif]

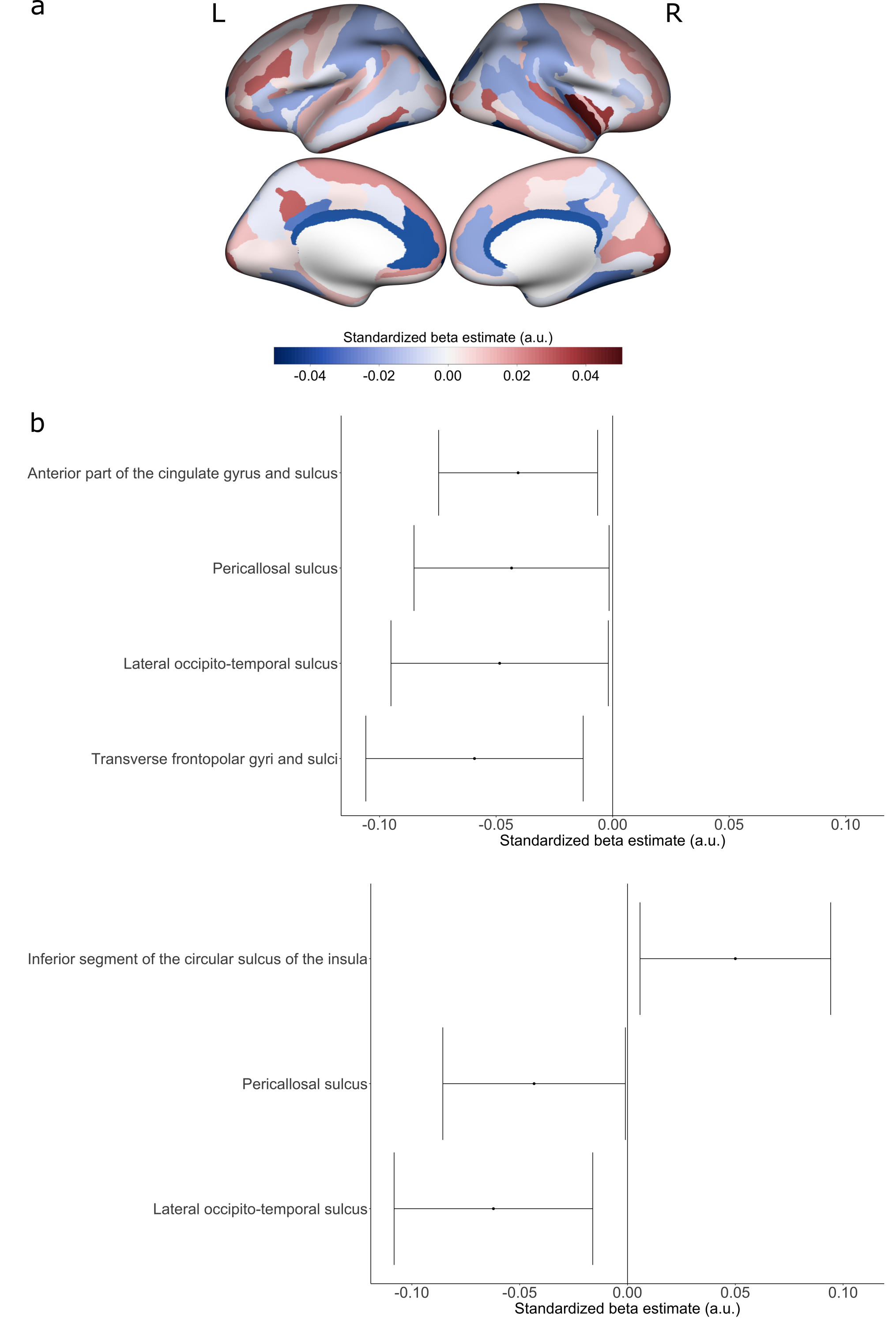

Supplement: Extended Data Figure 4-3 — Preterm birth associations with cortical surface area in stringent quality subsample. a, Positive β estimates are shown in red, indicating greater surface area with shorter gestational age. Negative β estimates are shown in blue, indicating smaller surface area with shorter gestational age. b, Estimated standardized βs of cortical regions whose 99% confidence interval do not overlap 0 are displayed for (top) left hemisphere surface area and (bottom) right hemisphere surface area. a.u. = arbitrary units. Download Figure 4-3, TIF file. [file enu-eN-NWR-0196-22-s15.tif]

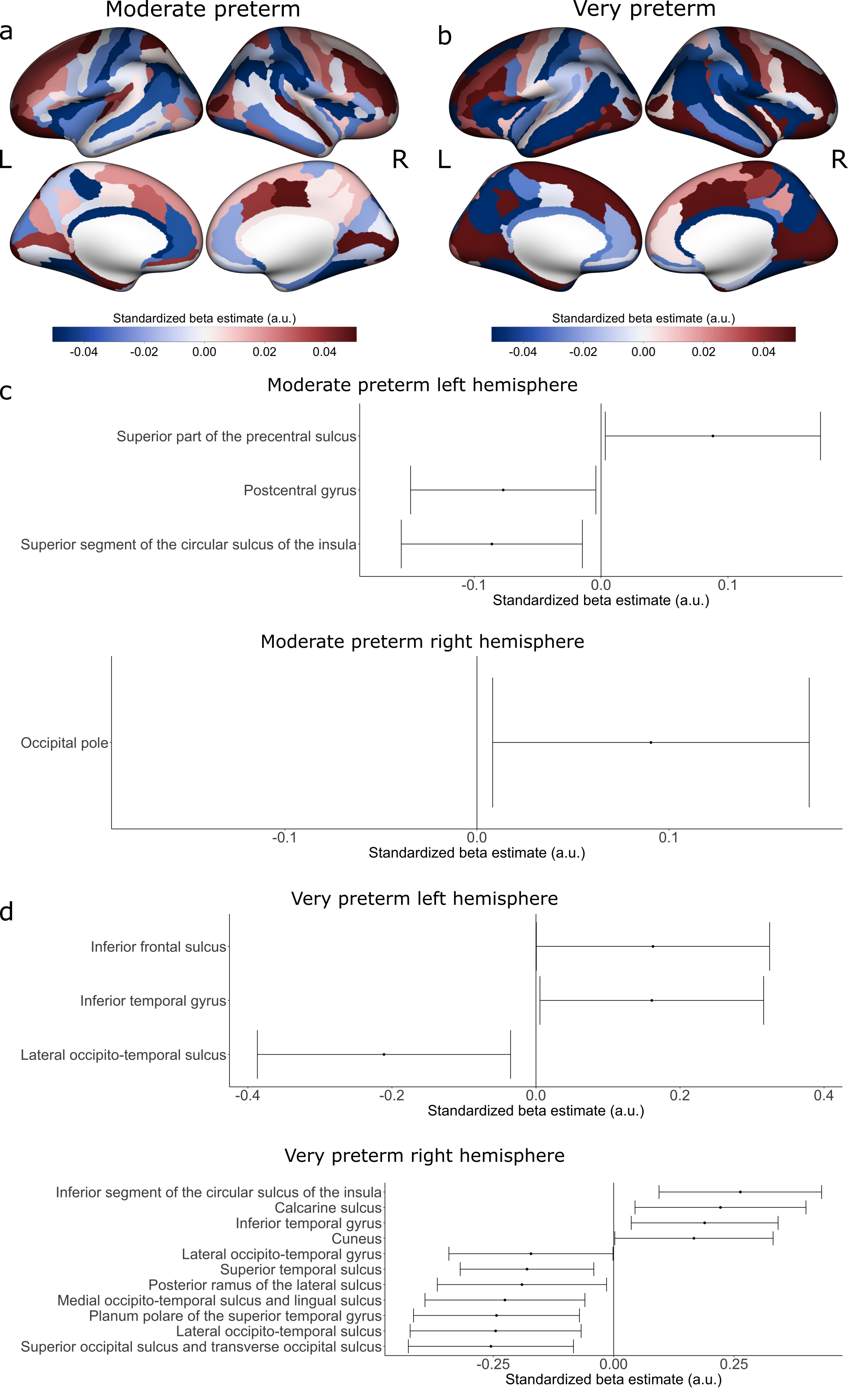

Supplement: Extended Data Figure 4-4 — Preterm birth associations with cortical surface area in moderate preterm birth relative to full term birth and very preterm birth relative to full term birth. Positive β estimates are shown in red, indicating greater surface area with shorter gestational age, and negative β estimates are shown in blue, indicating smaller surface area with shorter gestational age, for (a) moderate preterm birth relative to full term birth and (b) very preterm birth relative to full term birth. Estimated standardized βs of cortical regions whose 99% confidence interval do not overlap 0 are displayed for (top) left hemisphere surface area and (bottom) right hemisphere surface area for (c) moderate preterm birth relative to full term birth and (d) very preterm birth relative to full term birth. a.u. = arbitrary units. Download Figure 4-4, TIF file. [file enu-eN-NWR-0196-22-s17.tif]

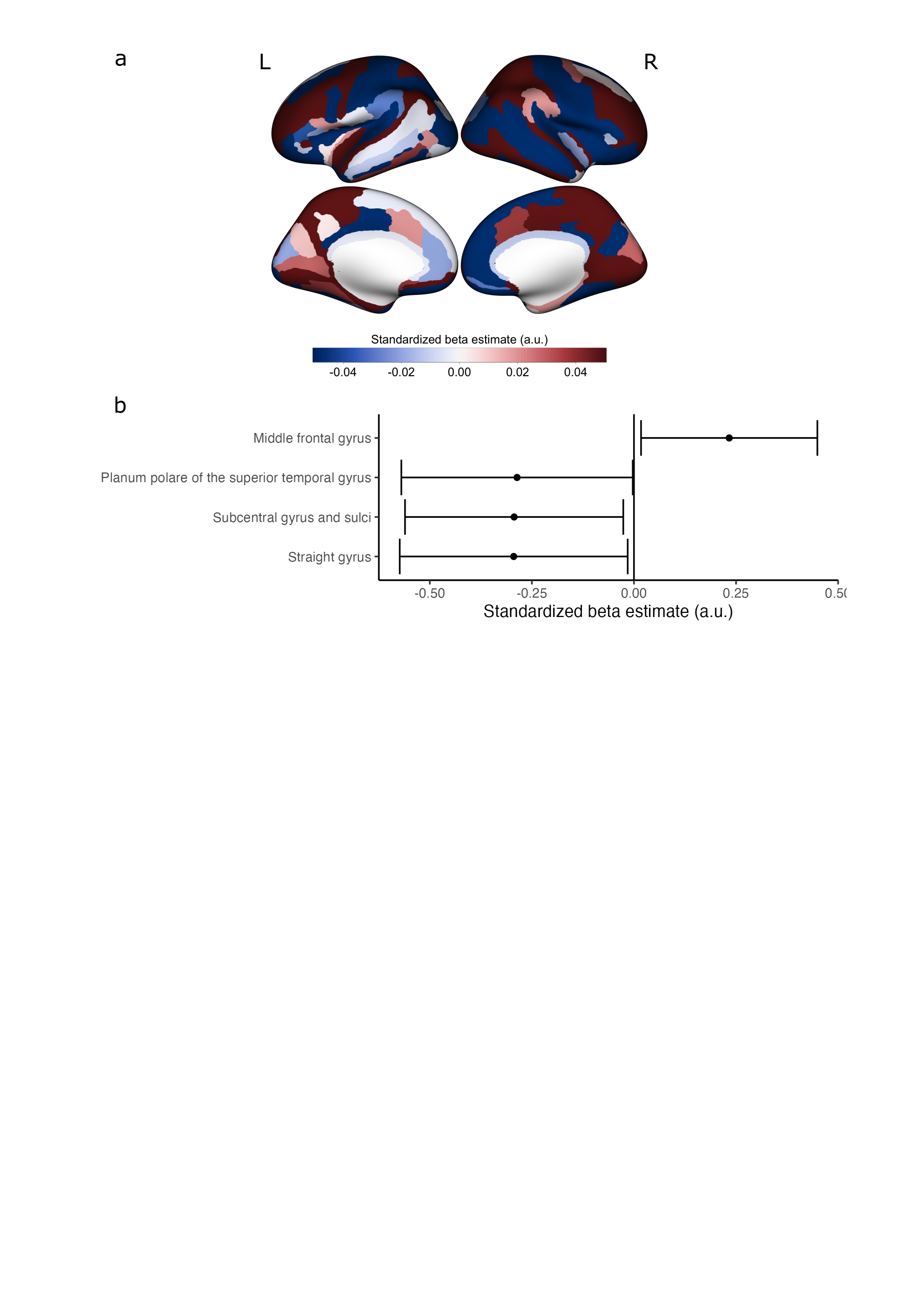

Supplement: Extended Data Figure 4-5 — Birth complication associations with cortical surface area. a, Positive β estimates are shown in red, indicating greater cortical surface area in children who experienced a birth complication requiring hospital stay. Negative β estimates are shown in blue, indicating smaller cortical surface area in children who experienced a birth complication requiring hospital stay. b, Estimated standardized βs of cortical regions whose 99% confidence interval do not overlap 0 are displayed for right hemisphere cortical surface area; all estimated standardized βs of cortical regions in the left hemisphere had a 99% confidence interval that overlapped 0. a.u. = arbitrary units. Download Figure 4-5, TIF file. [file enu-eN-NWR-0196-22-s10.tif]

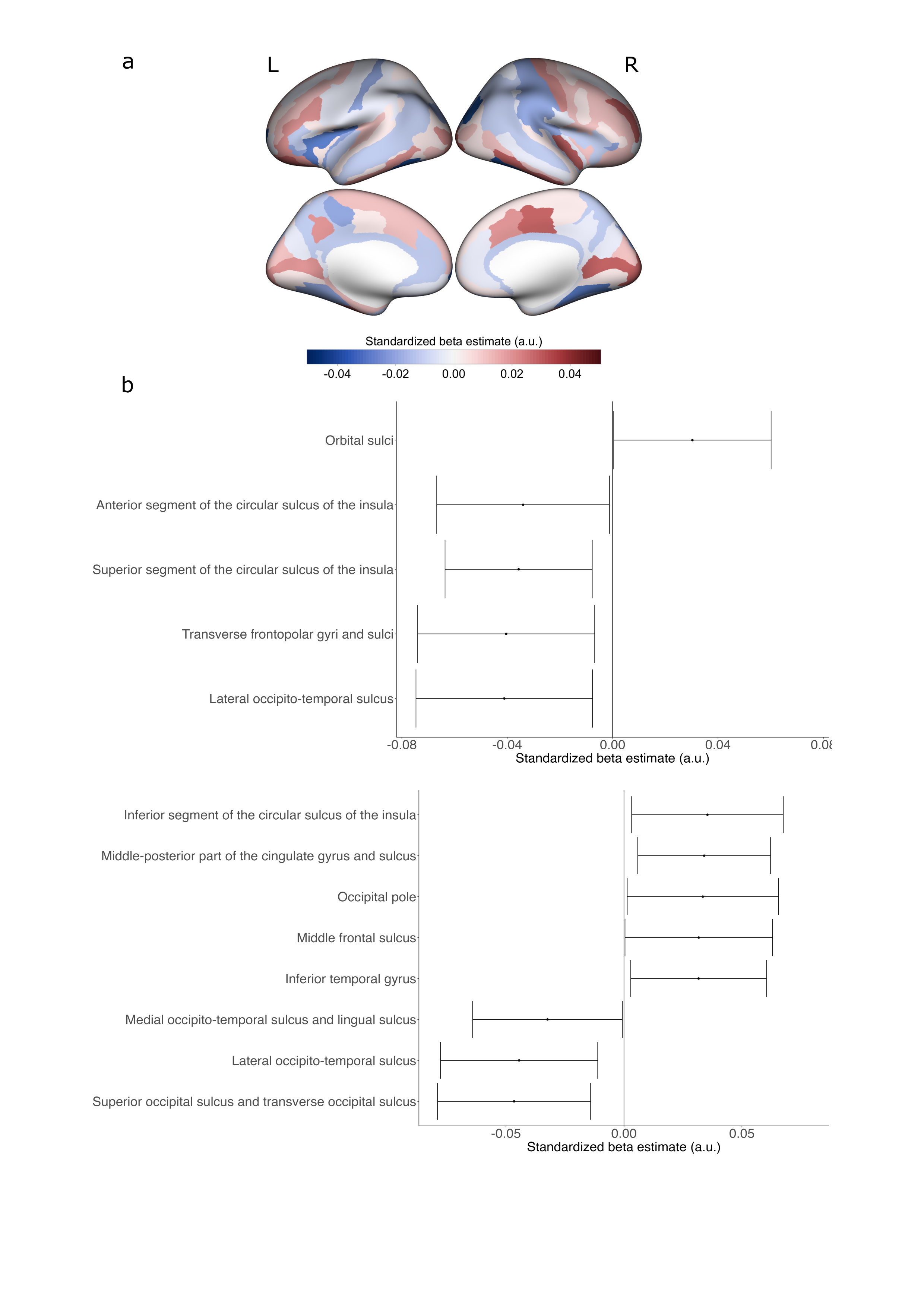

Supplement: Extended Data Figure 4-6 — Preterm birth associations with cortical surface area with a linear control for birth complications. a, Positive β estimates are shown in red, indicating greater cortical surface area with shorter gestational age. Negative β estimates are shown in blue, indicating smaller cortical surface area with shorter gestational age. b, Estimated standardized βs of cortical regions whose 99% confidence interval do not overlap 0 are displayed for (top) left hemisphere cortical surface area and (bottom) right hemisphere cortical surface area. a.u. = arbitrary units. Download Figure 4-6, TIF file. [file enu-eN-NWR-0196-22-s12.tif]

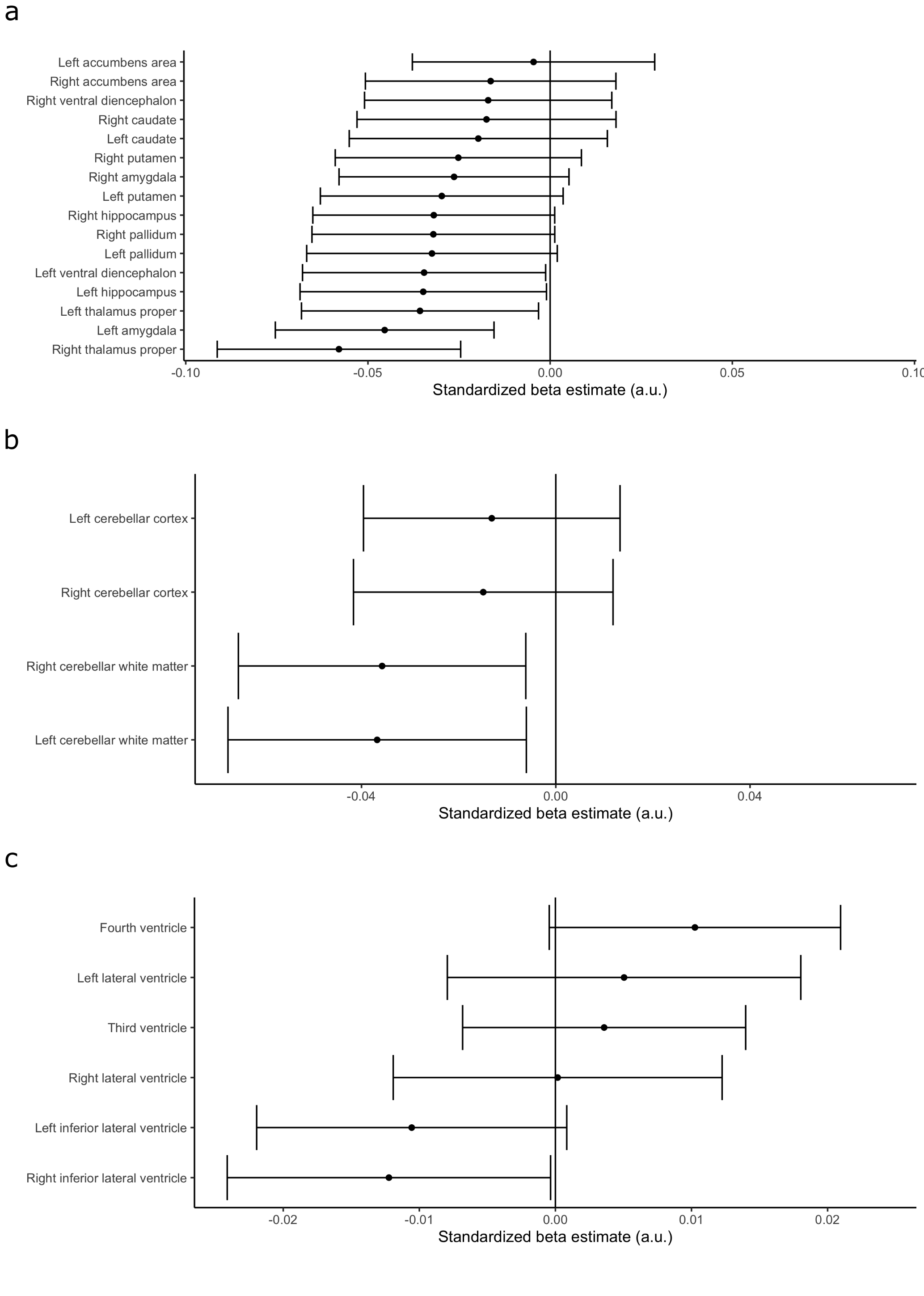

Supplement: Extended Data Figure 5-1 — Preterm birth associations with (a) subcortical, (b) cerebellar, and (c) log-transformed ventricular volumes without a control for total brain volume. Estimated standardized βs with 99% confidence intervals are shown. a.u. = arbitrary units. Download Figure 5-1, TIF file. [file enu-eN-NWR-0196-22-s18.tif]

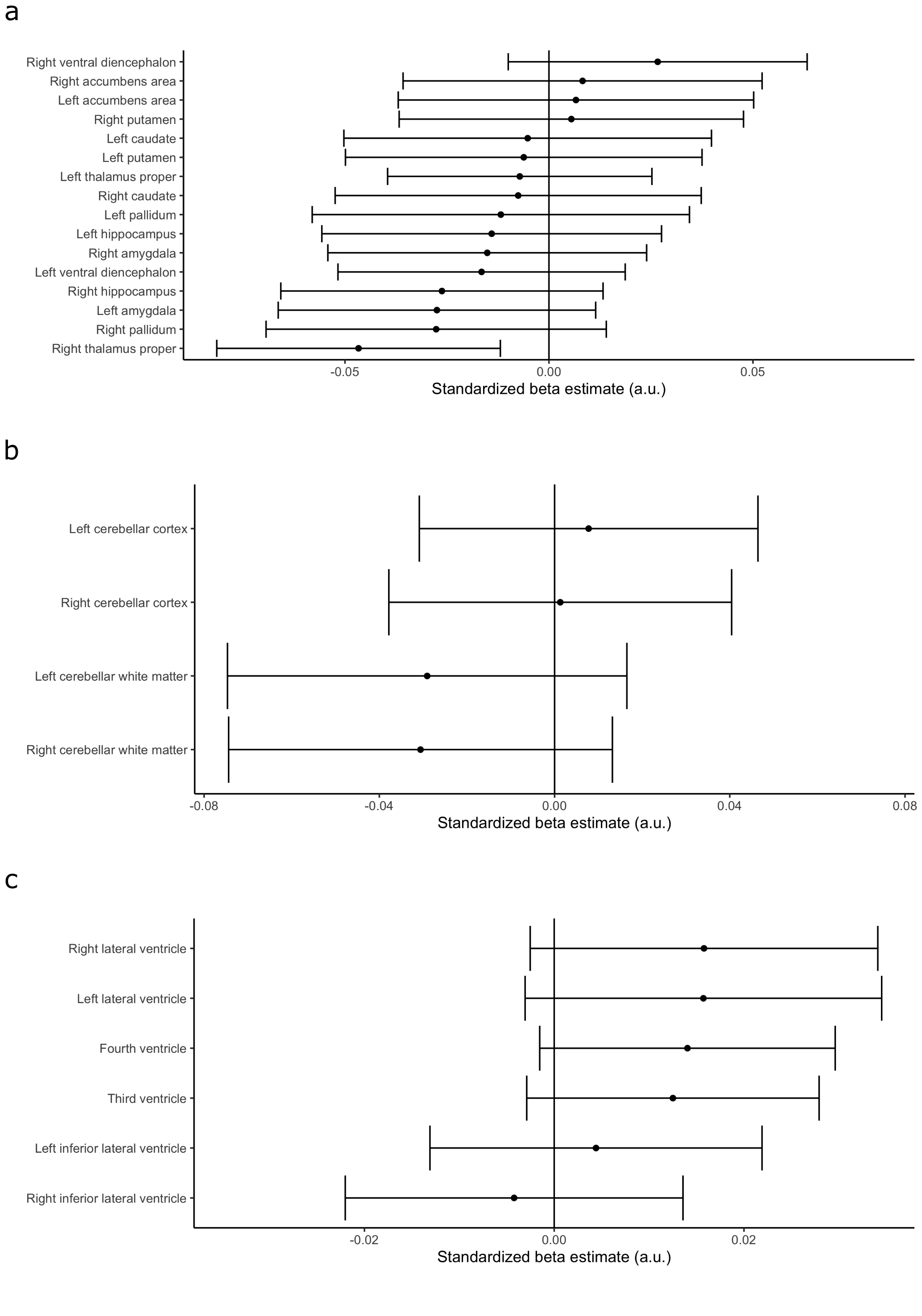

Supplement: Extended Data Figure 5-2 — Preterm birth associations with (a) subcortical, (b) cerebellar, and (c) log-transformed ventricular volumes in stringent quality subsample. Estimated standardized βs with 99% confidence intervals are shown. a.u. = arbitrary units. Download Figure 5-2, TIF file. [file enu-eN-NWR-0196-22-s19.tif]

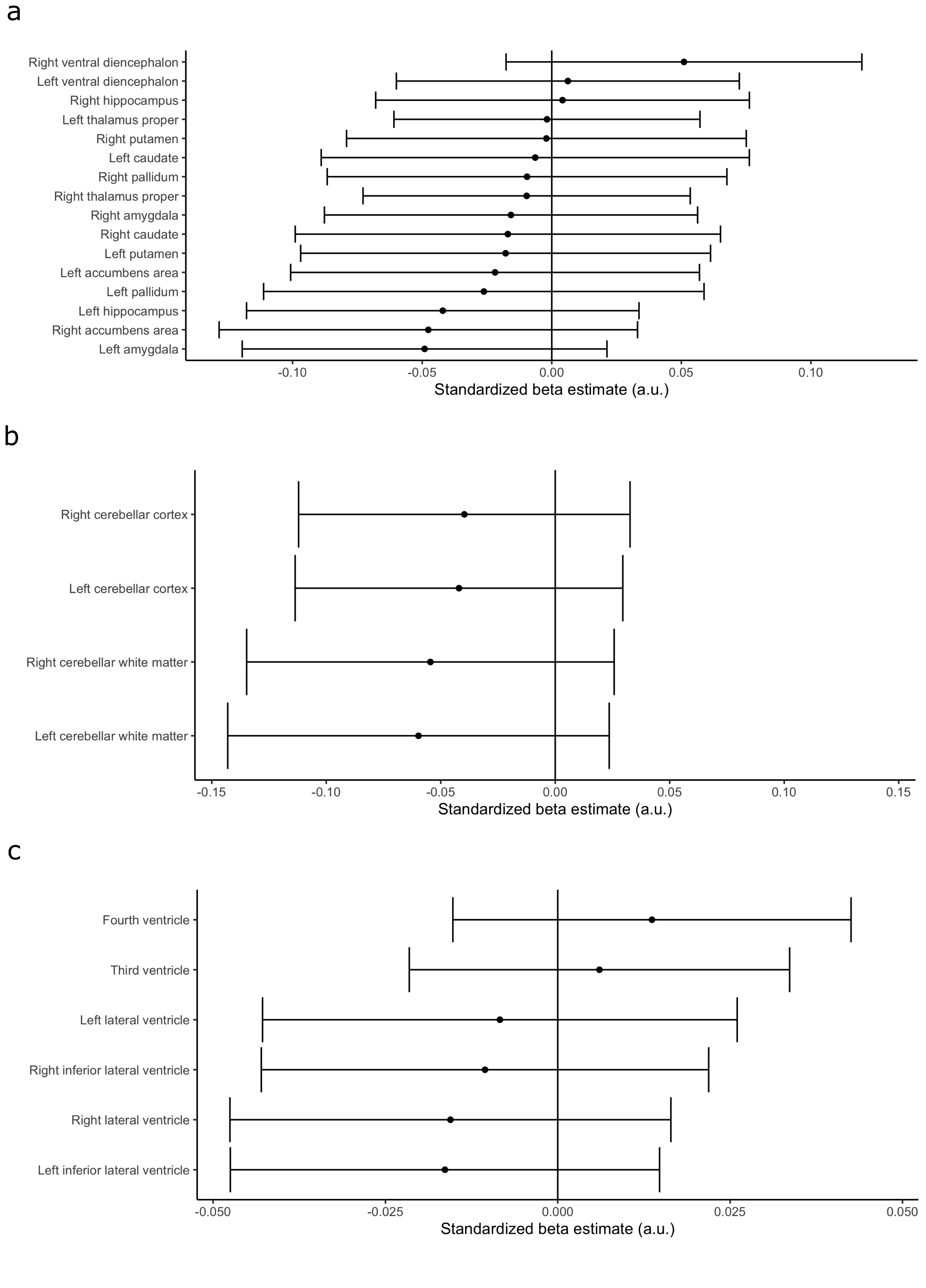

Supplement: Extended Data Figure 5-3 — Preterm birth associations with (a) subcortical, (b) cerebellar, and (c) log-transformed ventricular volumes in moderate preterm birth relative to full term birth. Estimated standardized βs with 99% confidence intervals are shown. a.u. = arbitrary units. Download Figure 5-3, TIF file. [file enu-eN-NWR-0196-22-s20.tif]

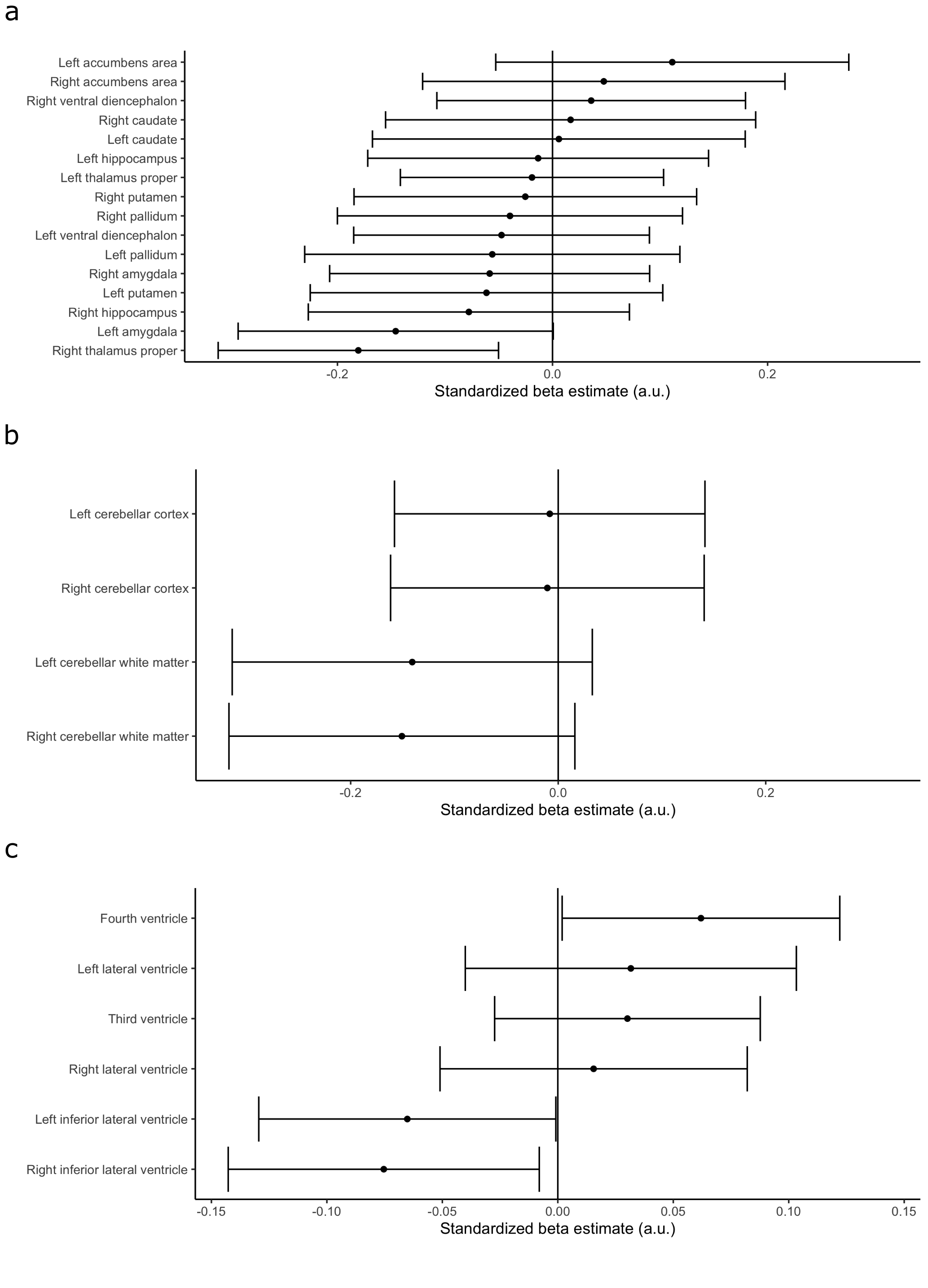

Supplement: Extended Data Figure 5-4 — Preterm birth associations with (a) subcortical, (b) cerebellar, and (c) log-transformed ventricular volumes in very preterm birth relative to full term birth. Estimated standardized βs with 99% confidence intervals are shown. a.u. = arbitrary units. Download Figure 5-4, TIF file. [file enu-eN-NWR-0196-22-s21.tif]

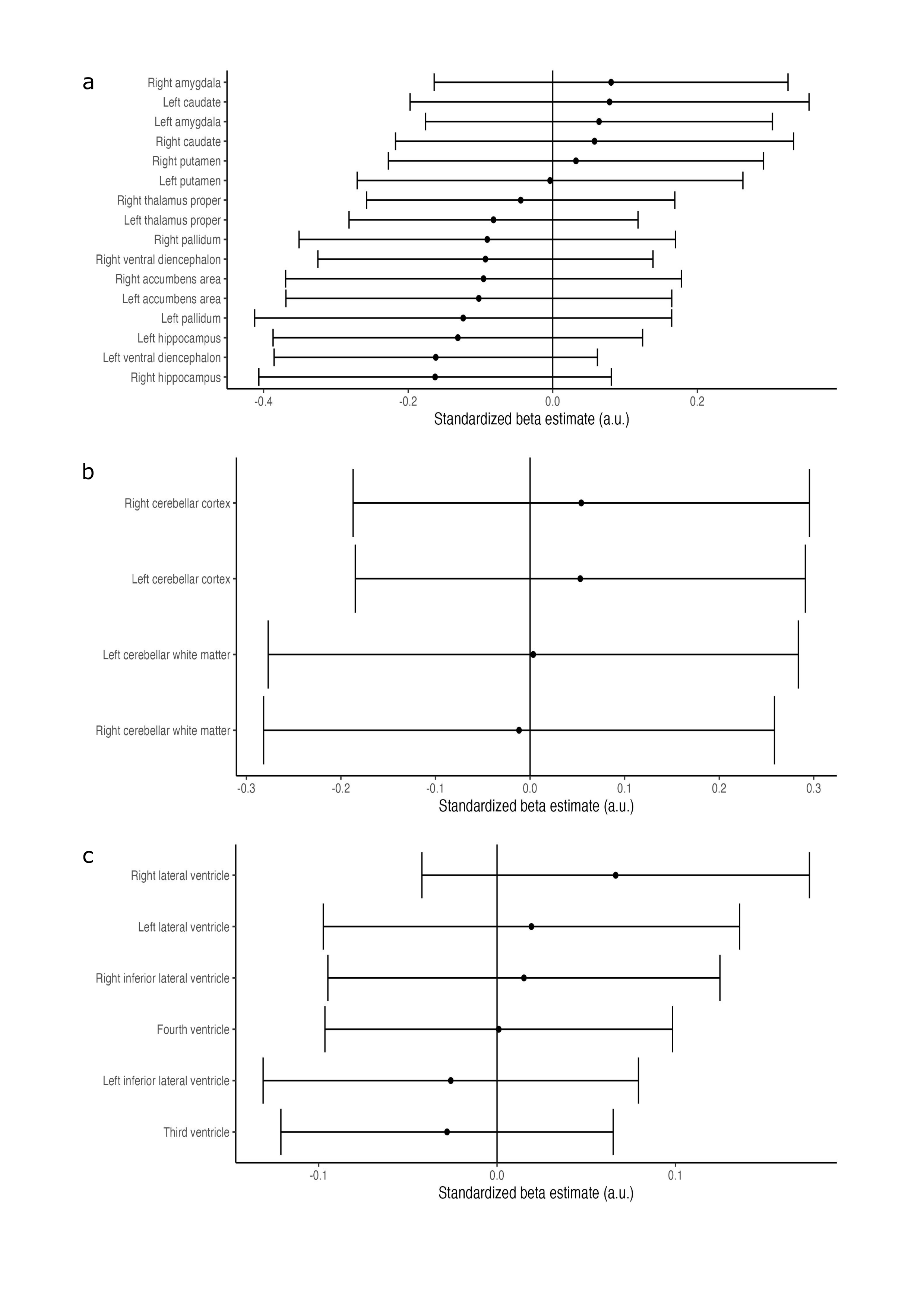

Supplement: Extended Data Figure 5-5 — Birth complication associations with (a) subcortical, (b) cerebellar, and (c) log-transformed ventricular volumes. Estimated standardized βs with 99% confidence intervals are shown. a.u. = arbitrary units. Download Figure 5-5, TIF file. [file enu-eN-NWR-0196-22-s14.tif]

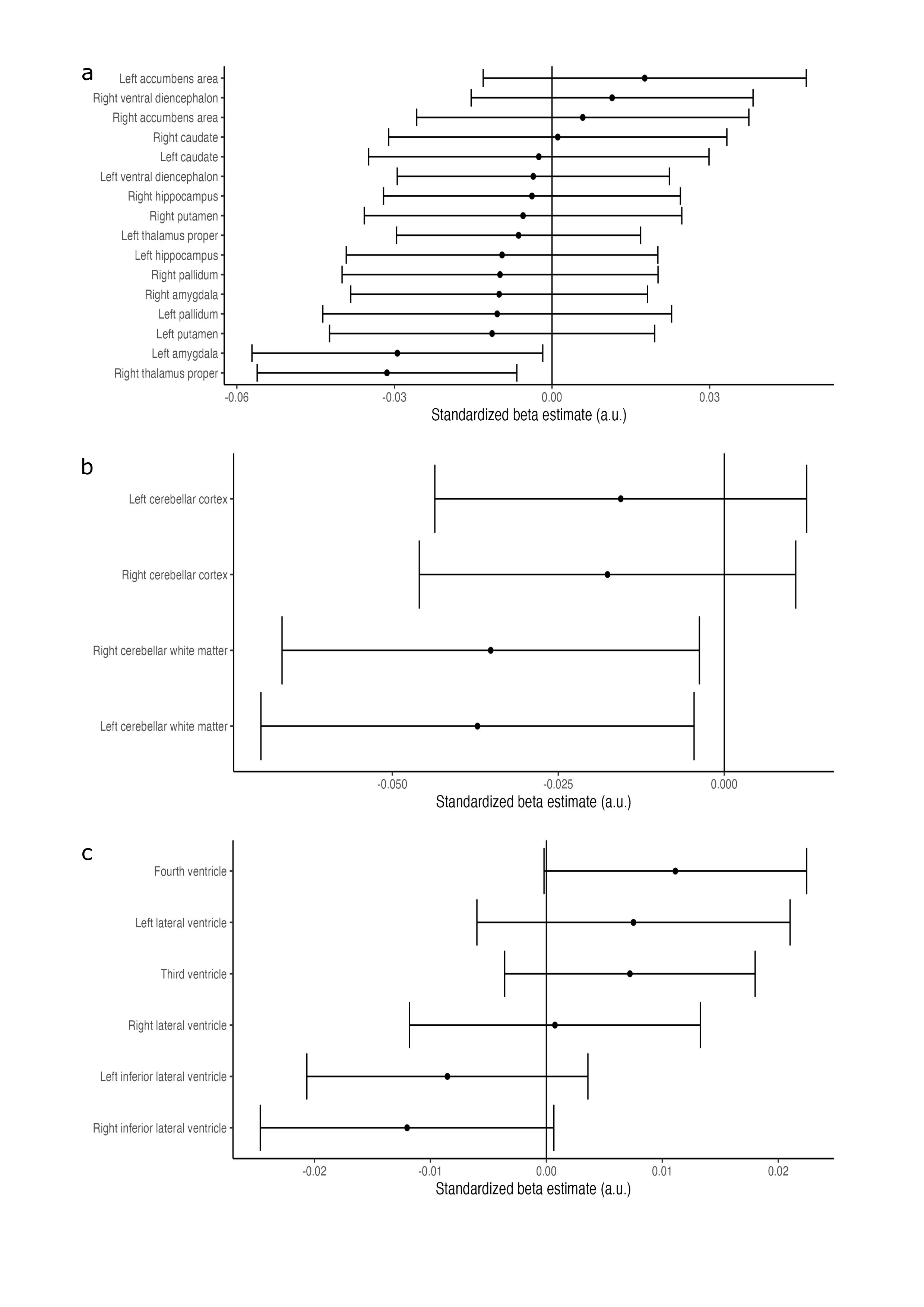

Supplement: Extended Data Figure 5-6 — Preterm birth associations with (a) subcortical, (b) cerebellar, and (c) log-transformed ventricular volumes with a linear control for birth complications. Estimated standardized βs with 99% confidence intervals are shown. a.u. = arbitrary units. Download Figure 5-6, TIF file. [file enu-eN-NWR-0196-22-s16.tif]

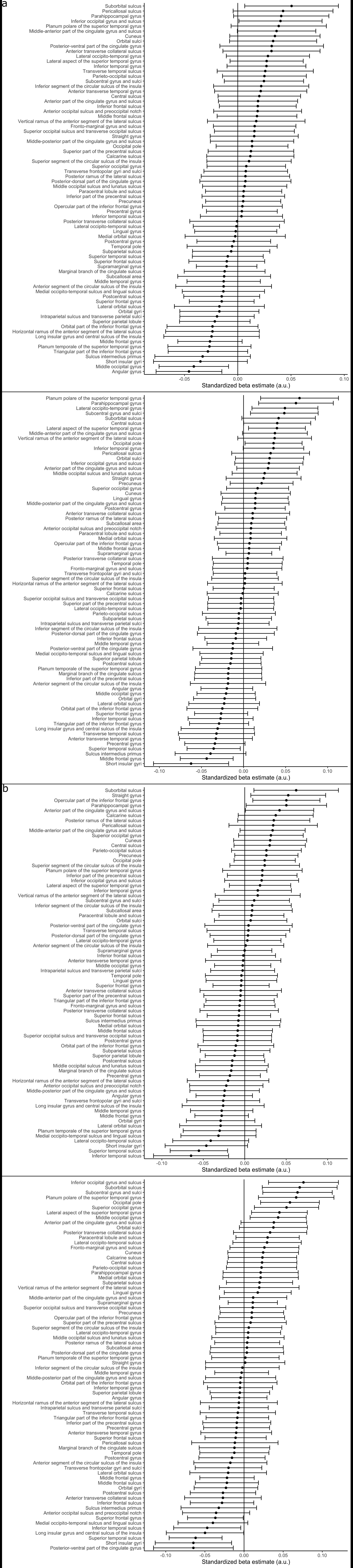

Supplement: Extended Data Figure 7-1 — Preterm birth associations with cortical thickness in (a) males and (b) females in all cortical regions. Estimated standardized βs are displayed with their 99% confidence intervals separately for the (top) left hemisphere cortical thickness and the (bottom) right hemisphere cortical thickness. a.u. = arbitrary units. Download Figure 7-1, TIF file. [file enu-eN-NWR-0196-22-s22.tif]

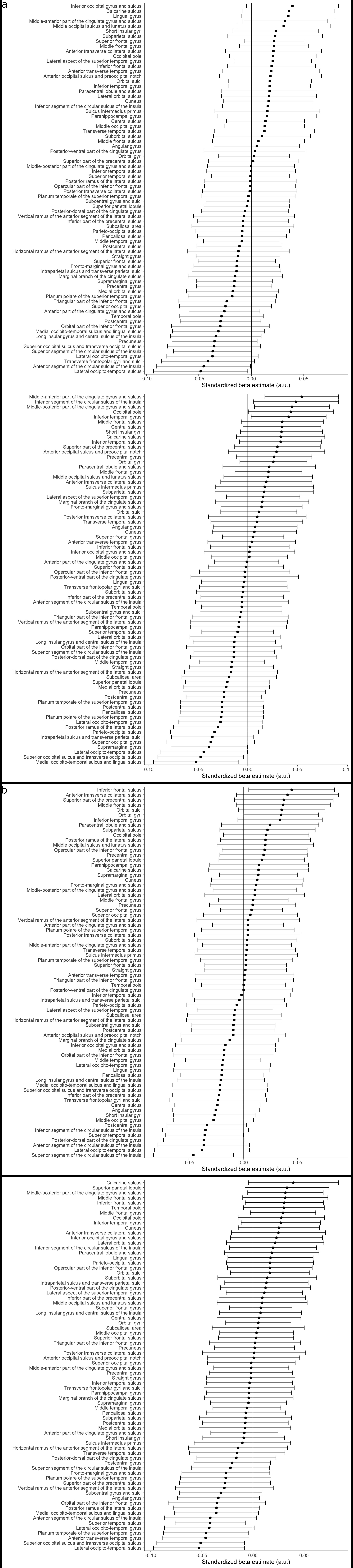

Supplement: Extended Data Figure 8-1 — Preterm birth associations with cortical surface area in (a) males and (b) females in all cortical regions. Estimated standardized βs are displayed with their 99% confidence intervals separately for the (top) left hemisphere surface area and the (bottom) right hemisphere surface area. a.u. = arbitrary units. Download Figure 8-1, TIF file. [file enu-eN-NWR-0196-22-s23.tif]
